# Genome-Wide Identification of Two-Component System Genes in Cucurbitaceae Crops and Expression Profiling Analyses in Cucumber

**DOI:** 10.3389/fpls.2016.00899

**Published:** 2016-06-22

**Authors:** Yanjun He, Xue Liu, Tao Zou, Changtian Pan, Li Qin, Lifei Chen, Gang Lu

**Affiliations:** ^1^Key Laboratory of Horticultural Plant Growth, Development and Biotechnology, Agricultural Ministry of China, Department of Horticulture, Zhejiang UniversityHangzhou, China; ^2^Zhejiang Provincial Key Laboratory of Horticultural Plant Integrative BiologyHangzhou, China

**Keywords:** two-component system, *Cucumis sativus* L., *Citrullus lanatus*, phylogeny, evolution, expression profiles

## Abstract

Cucumber and watermelon, which belong to Cucurbitaceae family, are economically important cultivated crops worldwide. However, these crops are vulnerable to various adverse environments. Two-component system (TCS), consisting of histidine kinases (HKs), phosphotransfers (HPs), and response regulator proteins (RRs), plays important roles in various plant developmental processes and signaling transduction in responses to a wide range of biotic and abiotic stresses. No systematic investigation has been conducted on TCS genes in Cucurbitaceae species. Based on the completion of the cucumber and watermelon genome draft, we identified 46 and 49 TCS genes in cucumber and watermelon, respectively. The cucumber TCS members included 18 HK(L)s, 7 HPs, and 21 RRs, whereas the watermelon TCS system consisted of 19 HK(L)s, 6 HPs, and 24 RRs. The sequences and domains of TCS members from these two species were highly conserved. Gene duplication events occurred rarely, which might have resulted from the absence of recent whole-genome duplication event in these two Cucurbitaceae crops. Numerous stress- and hormone-responsive *cis*-elements were detected in the putative promoter regions of the cucumber TCS genes. Meanwhile, quantitative real-time PCR indicated that most of the TCS genes in cucumber were specifically or preferentially expressed in certain tissues or organs, especially in the early developing fruit. Some TCS genes exhibited diverse patterns of gene expression in response to abiotic stresses as well as exogenous trans-zeatin (ZT) and abscisic acid (ABA) treatment, suggesting that TCS genes might play significant roles in responses to various abiotic stresses and hormones in Cucurbitaceae crops.

## Introduction

Protein phosphorylation is a key mechanism for regulating signal transduction in eukaryotes and prokaryotes. A two-component system (TCS) via phosphorylation between histidine and aspartic-acid (His-Asp) residues has been widely reported in prokaryotes (Stock et al., 2000). A simple TCS system consists of a membrane-associated histidine protein kinase (HK) and a cytoplasmic response regulator (RR). HK protein senses environmental signals and autophosphorylates its histidine residue (H), then the phosphate is transferred to an aspartate residue (D) of RR protein (Stock et al., 2000; Urao et al., 2000; Hwang et al., 2002). A complex TCS signaling system has been identified in some eukaryotic species, including higher plants (Urao et al., 2000). Plant TCS components typically consist of three signal transducers, namely hybrid HKs with both a His-kinase (HK) domain and a receiver (Rec) domain, histidine-containing phosphotransfers (HPs), and RRs. HP is regarded as a medium for the transfer of the phosphoryl group between the HK and the RR (Urao et al., 2000; Hwang et al., 2002; Schaller et al., 2008). TCS signal transduction mechanism was deeply investigated in plant cytokinin signaling (Hwang et al., 2002; Grefen and Harter, 2004). In *Arabidopsis*, AHK2, AHK3, and AHK4 function as cytokinin receptors and respond to stress negatively in cytokinin signal via TCS. These cytokinin receptors perceived cytokinin and were autophosphorylated. Then AHPs received the phosphate group from HKs and transferred to the Rec domain of type-B RRs. Finally, type-B ARRs activated type-A ARRs from putative repressors in the nucleus. Additionally, type-B ARRs could bind to multiple *cis*-elements in the promoter of target genes, such as MAPKs and other stress-related genes, to participate in stress responses (Wurgler-Murphy and Saito, 1997; Hwang et al., 2002; Grefen and Harter, 2004).

TCS pathway is one of the most important mechanisms for stress signal transduction, such as drought, high salinity, and inappropriate temperature. Almost all of HKs and HPs in *Arabidopsis* negatively or positively respond to salt, drought, and cold stresses (Tran et al., 2007, 2010; Wohlbach et al., 2008; Pham et al., 2012; Jeon and Kim, 2013; Kumar et al., 2013). *Arabidopsis AHP1, AHP2*, and *AHP3* genes are markedly downregulated by heat stress (Miyata et al., 1998). All type-A ARRs participate in the osmosis-related stresses by interacting with abscisic acid (ABA) with positive or negative response. Many RR genes are also induced by drought, high salinity, and low temperature (Wohlbach et al., 2008; Jeon et al., 2010). Additionally, a set of TCS elements in other crops have been proved to participate in abiotic stresses. Rice *OsAHP1/2* silencing seedlings oppositely respond to salt treatment and drought stress (Sun et al., 2014). OsHK3 is involved in ABA-induced antioxidant defense (Wen et al., 2015). Most of soybean TCS genes could negatively respond to dehydration stress (Le et al., 2011). Some tomato TCSs are involved in modulating drought stress responses (D'Amico-Damião et al., 2015). The tomato pollens in the ethylene receptor mutant *LE-ETR3 (Nr)* which is a member of HK family are more sensitive to heat stress via affecting pollen carbohydrate metabolism (Firon et al., 2012).

Cucumber (*Cucumis sativus* L.) and watermelon (*Citrullus lanatus*), which belong to the Cucurbitaceae family, are economically important crops consumed worldwide. However, these crops are highly susceptible to various adverse environments, such as inappropriate temperature, drought, and pathogens. TCS play essential roles in plant stress signaling network. Thus, applying a systematic identification and functional study of these stress responsive genes is necessary to elucidate the molecular mechanisms of tolerance and susceptibility in Cucurbitaceae crops (e.g., cucumber and watermelon). So far, few TCS genes have been characterized in Cucurbitaceae crops (Yamasaki et al., 2000; Karakurt et al., 2014). Three ethylene receptors in cucumber belonged to HK(L) family, have been proved to participate in the formation and development of female flower (Yamasaki et al., 2000, 2003; Wang et al., 2010). Cucumber PHYB, a HKL member, plays important roles in the response to cold tolerance (Sysoeva et al., 2013). Complete cucumber and watermelon genome drafts facilitate the exploitation of novel bioinformatics tools to identify and analyze the key elements of the TCS pathway. In this investigation, all putative TCS elements in cucumber and watermelon were identified *in silico* study. The classification, gene structures, conserved domains, chromosome distribution, phylogenetic relationship, synteny relationship, and gene duplication events of the TCS gene families were predicted and analyzed in detail. Stress- and hormone-responsive *cis*-elements were detected in the putative promoter regions of the TCS genes. Finally, the expression profiles of cucumber TCS genes in different plant organs/tissues and as responses to different abiotic stresses and plant hormones were examined by quantitative real-time PCR (qRT-PCR). Comprehensive analysis of TCS elements in Cucurbitaceae species provided insights into the structure and evolution of this system. Moreover, the results elucidated the potential roles of the TCS elements in stress and hormone response, which would provide a framework for future functional dissection of TCS in plant hormone and stress signal transduction.

## Materials and methods

### Identification of TCS genes in cucumber and watermelon

Protein sequences of all known TCS genes, particularly, 56, 52, 51, 62, 98, and 85 members from *Arabidopsis*, rice, maize, wheat, soybean, and Chinese cabbage, respectively, were downloaded from Phytozome (http://phytozome.jgi.doe.gov/pz/portal.html) and then used as queries to perform BLASTP searches with *E*-value of 1e^−5^ as the threshold (Mochida et al., 2010; Gahlaut et al., 2014). Meanwhile, Hidden Markov Model (HMM) profiles were generated using TCS conserved domain sequences from Pfam (http://pfam.janelia.org/), which were applied to identify the cucumber and watermelon TCS proteins using HMMER 3.0 software (http://hmmer.janelia.org/) with a default *E*-value. Subsequently, redundant sequences were omitted to retain unique TCS genes. Finally, the putative TCS members were further confirmed using SMART databases (http://smart.embl-heidelberg.de/) according to whether these members possess the structural characteristics and conserved domains of TCS elements. These domains included HisK domain, HATPase domain, receiver domain (Rec), CHASE domain, ethylene-binding domain (C2H4), His-containing phosphotransfer domain (HPt), and pseudo-HPt domain. Watermelon TCS members were identified using the same method against the watermelon proteome data set. Finally, all identified sequences were checked in *Arabidopsis* databases of TAIR website (http://www.arabidopsis.org/) to explore their homolog genes with the highest score.

Nucleotide sequences of all TCS genes were used as queries to perform BLASTN searches against the cucumber chromosomes (http://www.icugi.org/cgibin/ICuGI/index.cgi). The positions of these genes in cucumber and watermelon genomes were then obtained. ExPASy (http://web.expasy.org/compute_pi/) was used to calculate molecular weights and isoelectric points (PIs) of putative TCS proteins of cucumber and watermelon. Subcellular localizations were predicted using TargetP website (http://www.cbs.dtu.dk/services/TargetP/).

### Gene structure construction, motif analysis, and phylogenetic analysis

The structures of all cucumber and watermelon TCS genes were analyzed using the Gene Structure Display Server (http://gsds.cbi.pku.edu.cn/). MEME (http://meme.nbcr.net/meme/intro.html) was used for motif analysis to annotate the conserved motifs in these TCS proteins. The predicted peptide sequences of conserved domain in the TCS proteins were identified using the SMART database (http://smart.embl-heidelberg.de/). Then, multiple-sequence alignment for the predicted peptide sequences of conserved domains [HK(L) domain, Rec domain, and HPt or pseudo-HPt domain] was generated using Clustal X v1.81 with default parameters (Thompson et al., 1997). Similarity of the TCS genes from *Arabidopsis*, rice, cucumber, and watermelon was calculated by DNAStar software (Madison, WI). Phylogenetic analysis was performed using MEGA 5.0 program by neighbor-joining (NJ) method with 1000 replicates of the bootstrap based on the full-length protein sequences (Tamura et al., 2011).

### Chromosomal localization and evolutionary analysis of TCS genes

All TCS genes were assigned to corresponding cucumber or watermelon chromosomes based on Cucurbit Genomics Database. Tandem duplicated events were determined by identifying whether gene pairs were separated by fewer than five intervening genes and if they shared ≥40% amino acid sequence similarity (Hu and Liu, 2012). PGDD (http://chibba.agtec.uga.edu/duplication/) was used to perform synteny analysis and detect the homologous genes in different synteny regions and segment duplications, as described in cucumber MADS gene family (Hu and Liu, 2012). PGDD was also used to estimate the synonymous (*Ks*) and non-synonymous (*Ka*) substitution rates (Tang et al., 2008). CLUSTALW (http://www.genome.jp/tools/clustalw/) was used to align the amino-acid sequences and corresponding CDS sequences of TCS elements, and then *Ks* and *Ka* were calculated using the Codeml procedure of the PAML online program (http://www.bork.embl.de/pal2nal/). Divergence time of the duplicated genes and orthologous gene pairs between cucumber and watermelon were estimated using synonymous mutation rate of substitutions per synonymous site per year, as follows: T = *Ks*/2x (*x* = 6.56 × 10e^−9^; Lynch and Conery, 2000; Wang et al., 2015).

### Analysis of putative promoter regions of TCS genes in cucumber and watermelon

To investigate *cis*-elements in promoter sequences of TCS genes in cucumber and watermelon, 1.5 kb of genomic DNA sequences upstream of the initiation codon were obtained from Phytozome (http://phytozome.jgi.doe.gov/pz/portal.html). PlantCARE website (http://bioinformatics.psb.ugent.be/webtools/plantcare/html/search_CARE.html) was adopted to identify *cis*-elements in the promoter regions.

### Cucumber plant growth and treatments

Cucumber cv. jinglv, which is widely planted as a spring variety in China, was used for expression analysis. The plants were grown in a growth chamber in temperature-controlled greenhouses of Zhejiang University under day/night temperatures of 28/20±1°C and light intensity of 200 μmol m^−2^ s^−1^ with 16-h day length. The roots, stems, leaves, male flower buds (~1.0 cm in length), and female flower buds (~3.0 cm in length; Bai et al., 2004) were collected during the flowering period. Cucumber fruits were collected at 0, 3, and 9 days after pollination.

Three-week-old cucumber seedlings were used for abiotic stress and hormone treatments. Nutrient solution was supplied with 100 mM NaCl for salt treatment. Total roots were separately collected at 0, 1, 2, 4, and 8 h after treatments. For drought treatment, total roots were collected from 4, 6, and 8 days after the seedlings withholding water. For high- and low-temperature treatments, the seedlings were transferred to growth chambers at 35 ± 1°C and 4 ± 1°C, respectively, and the second true leaf leaves were sampled at 0, 1, 2, 4, and 8 h after high and low temperature treatments. To examine the expression profiles of TCS genes responding to cytokinin and ABA, 100 μM ZT and 100 μM ABA containing 0.05% Tween-20 were prepared and sprayed onto cucumber seedlings until the fully expanded leaves were covered with these solutions. The second true leaf on each plant was collected at 0, 1, 2, 4, and 8 h after treatments. All samples were collected in three biological replicates with 20 seedlings each. All plant materials were stored at −75°C until RNA isolation.

### RNA isolation and real-time PCR analysis

Total RNA was extracted from the collected materials using TRIZOL reagent (Invitrogen, Germany) according to the manufacturer-recommended protocol. The first cDNA strand was generated using the PrimerScript RT reagent kit (Takara, Japan) according to the manufacturer's instructions. Specific primers used in the qRT-PCR were designed by Primer 5 Software, and each primer was checked against cucumber DNA database to ensure its specificity.

The qRT-PCR reactions were performed on the CFX96 Real Time System machine (Bio-RAD, USA), programmed to heat for 30 s at 95°C, followed by 40 cycles of 5 s at 95°C and 45 s at 55°C, and at the end, 1 cycle of 1 min at 95°C, 30 s at 50°C, and 30 s at 95°C. Two biological and three technical replicates for each sample were performed with 15 μL of reaction volume using the SYBR Premix Ex Taq kit (TOYOBO, Japan). The *EF1a* gene (accession number EF446145) of *C. sativus* was selected as an internal control (Wan et al., 2010). Relative gene expression was calculated using the 2−^ΔΔCt^ method. Heatmap was generated by Multiple Array Viewer using the relative expression data of each gene.

## Results and discussion

### Identification of TCS proteins in cucumber and watermelon

BLASTP searches were performed in Cucurbit Genomics Database to explore the putative TCSs in cucumber and watermelon by using 280 TCS protein sequences as queries from *Arabidopsis*, rice, maize, soybean, wheat, and Chinese cabbage. A total of 236 protein hits including 80 HK(L)s, 44 HPs, and 112 RRs, were identified in the cucumber genome database. Additionally, 53 HK(L)s, 7 HPs, and 42 RRs were predicated using the HMMER 3.0 program hmmsearch with an default value. After the redundant sequences were omitted to obtain unique putative TCS genes, the remaining hits were further filtered using Pfam and SMART according to the presence of structural characteristics and conserved domains of TCS elements. Finally, 46 TCS members consisting of 18 HK(L)s, 7 HPs, and 21 RRs were identified in cucumber (Table S1). A similar approach was implemented to identify watermelon TCS genes, and 19 HK(L)s, 6 HPs, and 24 RRs were identified from the watermelon genomics database (Table S2). All cucumber and watermelon TCS members were named according to homology with *Arabidopsis* genes. This nomenclature was widely used in soybean (Mochida et al., 2010) and Chinese cabbage (Liu et al., 2014).

TCS genes had been intensively studied in some model plant species and important crops, such as *Arabidopsis* (Hwang et al., 2002), *Physcomitrella patens* (Ishida et al., 2010), *Lotus japonicas* (Ishida et al., 2009), rice (Pareek et al., 2006), maize (Chu et al., 2011), wheat (Gahlaut et al., 2014), soybean (Mochida et al., 2010), and Chinese cabbage (Liu et al., 2014). The numbers of known TCS genes in plant species are summarized in Table 1. Only 46 and 49 members were found in cucumber and watermelon, respectively, which were fewer than that in the reported plant species except wheat, *L. japonicas*, and *P. patens*. Small gene family sizes were also found in other gene families in cucumber genome (Ling et al., 2011). We speculated that this phenomenon resulted from the small genome size and fewer duplication events in the cucumber genome (Huang et al., 2009). Interestingly, cucumber genome evidently contained obviously fewer RRs than those of *Arabidopsis*, rice, soybean and other species. In particular, no type-C RR was found in cucumber. The reason for fewer RR gene members in cucumber is worthy of further investigation.

**Table 1:** **Summary of the TCS gene numbers identified in plants**.

**Species**	***HK(L)***	***HP (pseudo-HP)***	***Type-A RR***	***Type-B RR***	***Type-C RR***	***Pseudo RR***	**Total**	**References**
*Arabidopsis thaliana*	17 (9)	6 (1)	10	12	2	9	56	Schaller et al., 2008
*Oryza sativa*	11 (3)	5 (3)	13	13	2	8	52	Pareek et al., 2006
*Lotus japonicus*	14	7	7	11	1	5	40	Ishida et al., 2009
*Glycine max*	36 (15)	13	18	15	3	13	98	Mochida et al., 2010
*Zea mays*	11 (3)	9 (2)	16	9	3	11^a^	59	Chu et al., 2011
*Physcomitrella patens*	18	3	7	5	2	4	39	Ishida et al., 2010
*Triticum aestivum*	7	10	41	2	0	2	45	Gahlaut et al., 2014
*Brassica rapa*	11	8 (1)	21	17	4	15	85	Liu et al., 2014
*Cucumis sativus* L	18 (8)	7 (2)	8	8	0	5	46	This work
*Citrullus lanatus*	19 (9)	6 (2)	8	10	1	5	49	This work

a *Only clock PRR*.

### HK proteins in cucumber and watermelon

A total of 18 HK(L) proteins were identified in cucumber and classified into 10 CsHKs and 8 diverged CsHKLs according to whether they possessed conserved His-kinase transmitter (HK) domain (Table S1). HK proteins were further classified into four cytokinin receptor-like CsHKs, two AHK5-like CsHKs, two ethylene receptor-like CsHKs, one AHK1-like, and one CKI1-like CsHK. Cucumber HKs, except for CsHK3, usually have a typical HK domain with five conserved signature motifs, namely, H, N, G1, F, and G2, of which the conserved His site is the most critical feature (Figures S1, S2). Additionally, CsHKLs include five phytochrome (PHY)-like CsHKLs, two PDK-like CsHKLs, and one ETR2-like CsHKL.

CsHK1 shares 39% identity with its homolog CKI1 in *Arabidopsis* (Table S1), which has been proved to implicate in cytokinin signaling and regulate the development of female gametophytes in *Arabidopsis* (Kakimoto, 1996; Schaller et al., 2008). *CsHK2* and *CsHK3* are adjacent to each other on chromosome A4 (Figure S3). CsHK2 shares a high similarity of over 70% with CKI2 but a part of Rec domain is lost. The lost sequences may generate another protein CsHK3. These two genes could be spliced and form a conserved homologous CKI2-like gene with 61% identity (Figure S3). The event of protein domain loss also occurred in cucumber WRKY proteins (Ling et al., 2011).

**Figure 1:**
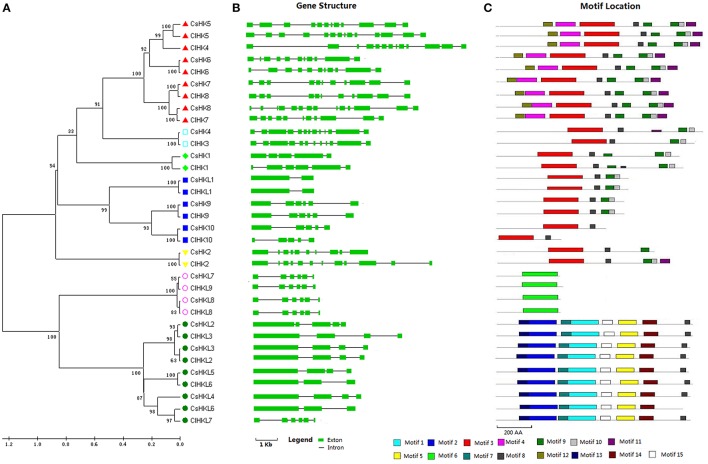
**Phylogenetic relationship, gene structures, and conserved motif of all HK(L) genes in cucumber and watermelon**. **(A)** The unrooted phylogenetic tree was generated based on the amino acid sequences by the neighbor-joining method using MEGA 5. Bootstrap supports from 1000 replicates are indicated at each branch. The different subgroup members were marked by different graphs. **(B)** Gene structure was analyzed using the Gene Structure Display Server online. The green boxes indicate the exons, and lines indicate the introns. **(C)** Motif analysis was performed using MEME 4.0 software as described in the Materials and methods. The different colored boxes represent different motifs in the corresponding position of each CsHK(L) proteins.

The cytokinin-receptor CsHKs including CsHK5-CsHK8 share high identities (63%-80%) with their counterparts AHK2-AHK4 in *Arabidopsis* (Figures 1, S14; Table S1). Domain analysis confirmed that they have seven conserved motifs including motifs 3, 4, and 8–12 identified using the MEME website (Figure 1C), and four type conserved domains, namely, HK, Rec, CHASE, and transmembrane (TM) domains identified using Pfam and SMART online tools (Figures S1, S2). CHASE domain, a cytokinin receptor characteristic domain, is crucial for recognizing and binding of cytokinin (Hwang et al., 2002). Additionally, one or two TM domains were found in these four cytokinin-receptor CsHKs, which have been proved to play important roles in membrane-associated signal recognition in plants (Pareek et al., 2006; Liu et al., 2014).

Three ethylene receptor members, namely, CsHK9, CsHK10, and CsHKL1, all have a characteristic C2H2 domain (Table S1), which is important for ethylene-binding (Hwang et al., 2002). Additionally, CsHK9 and CsHKL1 also contain three TM domains and a GAF (cyclic GMP adenylyl cyclase FhlA) domain (Figure S1). Contrary to CsHK9 and CsHK10, CsHKL1 protein contain a diverged HK domain and is homologous with *Arabidopsis* ETR2, which regulates seed germination during salt stress by modulating ABA signaling (Wilson et al., 2014).

Five PHY subfamily members, CsHKL2-CsHKL6, share 60–79% identities with their corresponding *Arabidopsis* counterparts. They all contain a HKL, a PHY (chromophore-binding), a GAF, and two PAS (signal sensor) domains (Figure S1, Table S1). PHY, GAF, and PAS domains play essential roles in responding to red and far-red light signals during plant development in *Arabidopsis* (Rockwell et al., 2006).

Similarly, 10 HK and 9 HKL members were identified in watermelon (Table S2). In detail, HK proteins include five cytokinin receptor ClHKs and two ethylene receptor ClHKs, as well as one CKI1, CKI2, and AHK1-like gene, respectively. The ClHKLs proteins contain six PHY-like ClHKLs, two PDK like ClHKLs, and one ETR2 like ClHKL (Table S2). The overall protein structure of ClHK(L)s members resemble to that in cucumber (Figures S4, S5).

### HP protein family in cucumber and watermelon

**Figure 2:**
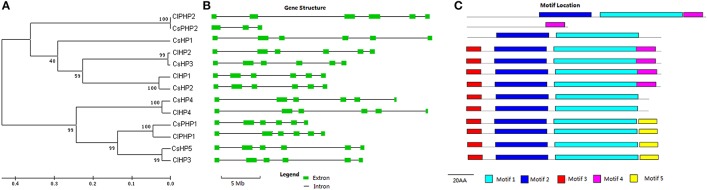
**Phylogenetic relationship, gene structures, and conserved motifs of the HP family members in cucumber and watermelon**. For the details, see Figure 1.

Seven HPs, which included five authentic HPs and two pseudo-HPs (PHPs) with a pseudo-HPt domain, were identified in the cucumber genome (Figures 2, S14; Table S1). The HP family elements, except CsHP1 and CsPHP2, have three conserved motifs, namely motif 1, motif 2, and motif 3 (Figure 2). Three AHP1-like members (CsHP1, CsHP2, and CsHP3) have 48–56% identities with their *Arabidopsis* homologs, whereas three AHP4-like members (CsHP4, CsHP5, and CsPHP1) share high identities (61–72%) to *Arabidopsis* AHP4. The CsHPs except for CsPHP1 and CsPHP2, have a conserved Hpt domain with a signature motif of XHQXKGSSXS (Hwang et al., 2002). The H sites in CsPHP1 and CsPHP2 are replaced by a Q and N residue, respectively (Figure S6). Notably, CsPHP1 has a pseudo-HPt domain but show higher sequence homology to the authentic-AHP4 than the pseudo-APHP1 in *Arabidopsis* (Table S1). Similar phenomenon was also found in rice, in which three pseudo-HPs (OsPHP1–OsPHP3) are closer to AHP4 than AHP6 in *Arabidopsis* (Pareek et al., 2006). Six HP proteins (four HPs and two pseudo-HPs) were identified in watermelon (Table S2, Figure S7). Similar to the cucumber HPs, the HP genes in watermelon have five introns, and their proteins, except for ClPHP2, all contain three conserved HP motifs (Figure 2).

### RR protein family in cucumber and watermelon

**Figure 3:**
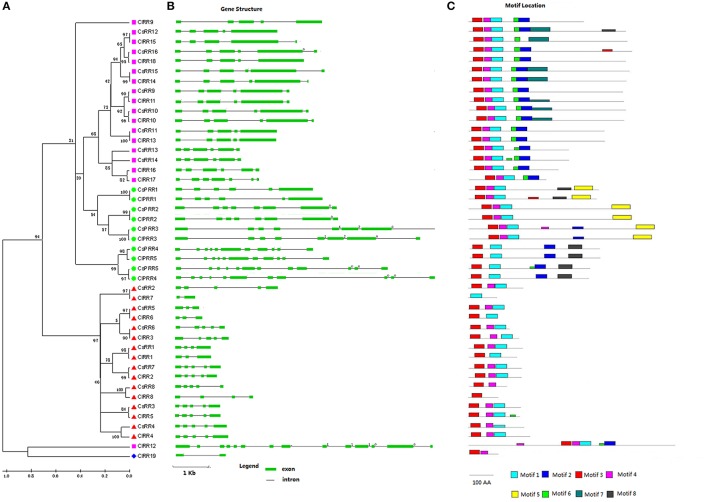
**Phylogenetic relationship, gene structures, and conserved motif of RR genes in cucumber and watermelon**. For the details, see Figure 1.

A total of 21 RRs in cucumber were identified including eight type-A RRs, eight type-B RRs, and five pseudo RRs (Table S1). Type-A RRs show 53–75% identity to their homologs in *Arabidopsis*. Protein analysis indicated that all eight type-A RRs had a receiver domain (Rec) corresponding to two conserved motif (motif 3 and 4) (Figures 3, S14). Eight type-B CsRRs (CsRR9-CsRR16), as transcription factors (TFs), are characterized with a long C-terminal extension containing a GARP domain (Figures S8, S9) which is a Myb-like DNA binding domain (Hwang et al., 2002). All type-B CsRRs have four or five introns, and their proteins contain five conserved motifs (motifs 1–4 and 6; Figure 3). In addition, five cucumber pseudo-RRs were further divided into Clock PRR and type-B PRR subgroups. Compared with authentic RRs, all PRRs have a pseudo-Rec domain, in which the conserved D sites are substituted by E amino acid residues. CsPRR1-CsPRR3 belonging to the Clock PRR, share 41–65% identity to their counterparts in *Arabidopsis*. These proteins are featured with a CCT domain (Figure S8) which plays important roles in regulating circadian rhythms (Niwa et al., 2007). *Arabidopsis* clock PRRs could control flowering time. CsPRR4 and CsPRR5 containing a Myb domain and pseudo-Rec domain were grouped into the type-B PRRs (Figures S7, S8). They show 45% and 41% identity to *Arabidopsis* APRR2, respectively. APRR2-like gene has been proved to participate in regulating fruit ripening and pigment accumulation in tomato and pepper (Pan et al., 2013).

Meanwhile, we identified 19 ClRRs in the watermelon genome. These ClRRs include 8 type-A RRs, 10 type-B RRs, 1 type-C RRs, and 5 PRRs (Table S2, Figures S10, S11). Interestingly, ClRR12, a type-B member, not only has a Rec and a Myb domain but also is infused with a TraB domain, which is a characteristic domain of TraB family proteins and TraB family members are pheromone shutdown-related proteins. This domain fusion phenomenon was also reported in Chinese cabbage (Liu et al., 2014).

### Phylogenetic relationship analysis of TCS members

**Figure 4:**
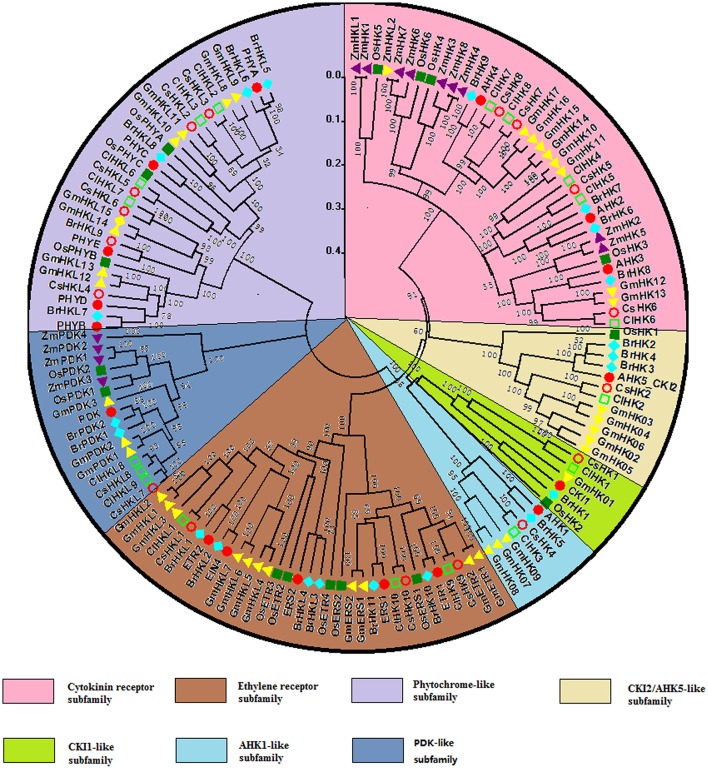
**Phylogenetic relationship of HK(L) proteins in ***Arabidopsis***, rice, maize, Chinese cabbage, soybean, cucumber, and watermelon**. The phylogenetic trees were constructed using the neighbor-joining method with bootstrap tests by MEGA 5.0. The bar represents the relative divergence of the sequences examined. Different subgroups of HK(L)s were highlighted by different colors.

To further evaluate the phylogenetic relationship of TCS genes, 142 HK(L)s, 54 HPs, and 248 RRs from *Arabidopsis* (Hwang et al., 2002), rice (Pareek et al., 2006), maize (Chu et al., 2011), soybean (Mochida et al., 2010), Chinese cabbage (Liu et al., 2014), cucumber, and watermelon were used to perform multiple alignments and construct phylogenetic trees. The HK(L)s phylogenetic tree indicated that all of HK(L) proteins in the seven species were divided into eight distinct subfamilies (Figure 4), namely cytokinin receptor, ethylene receptor, PHY-like, CKI1-like, CKI2/AHK5-like, AHK1-like, and PDK-like subfamily (Hwang et al., 2002; Schaller et al., 2008). Cucumber and watermelon HK(L)s have the nearest relationship with soybean except for CsHKL5 and ClHKL6. CKI2/AHK5-like and PDK-like subfamilies from dicots and monocots form two exclusive subgroups, respectively, which indicated that these HK(L)s might have formed after the divergence of monocotyledon and dicotyledon. To perform more comprehensive phylogenetic analysis of HK(L)s, we identified all PDK subfamily genes in the other six species. Most of PDK-like genes in the seven species share high similarity even over 90% with each other (Table S3) and they have only one HATPase domain (Figure S1). These demonstrated the PDK subfamily members kept highly conserved in protein sequences during evolution.

**Figure 5:**
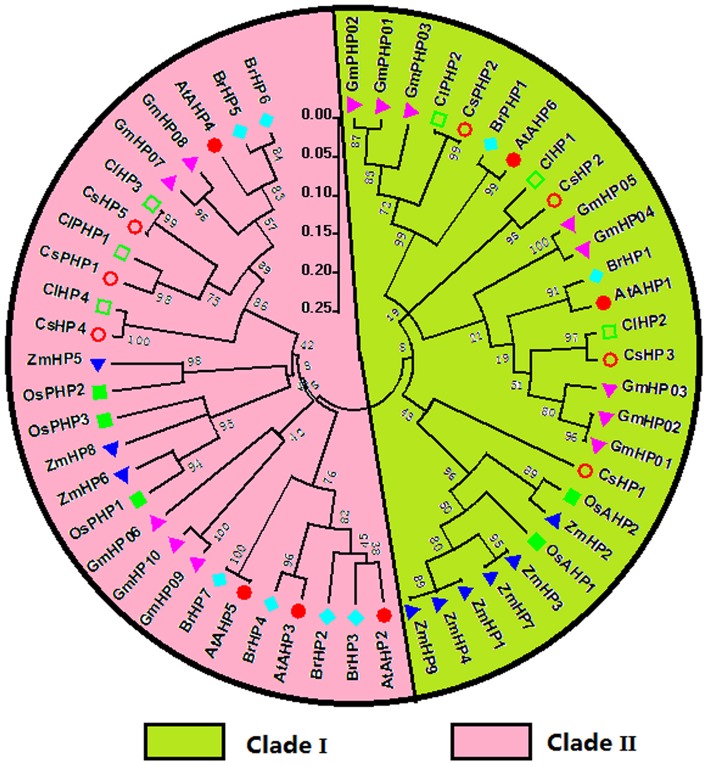
**Phylogenetic relationship of phosphotransfer (HP) proteins and related proteins in ***Arabidopsis***, rice, maize, Chinese cabbage, soybean, cucumber, and watermelon**. For the details, see Figure 4.

All the authentic and pseudo-HPs from these seven species as above mentioned were mainly divided into two clades, namely clade I and II (Figure 5). In the clade I, dicots HPs, except CsHP1, form a subclade distinct from that of monocots, which indicated this subgroup HPs expand prior to the monocot-eudicot divergence. CsHP1-3 and ClHP1-2 are closely related to AHP1, which functions in cytokinin signaling, and regulating root growth, vascular development, and seed set (Hutchison et al., 2006). In the clade II, the HPs from *Arabidopsis* and Chinese cabbage except AHP4 and BrHP4/5 form a distinct subclade with the other HPs from the other five species.

**Figure 6:**
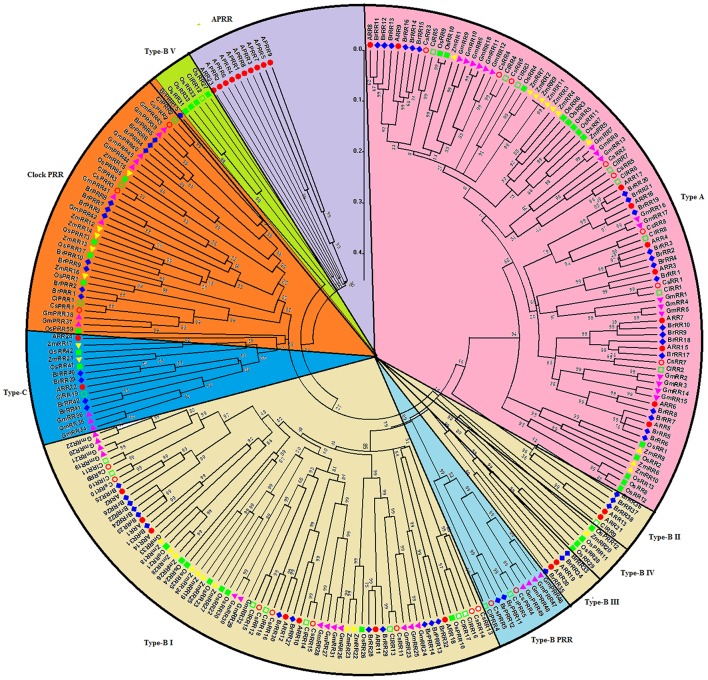
**Phylogenetic relationship of RR proteins in ***Arabidopsis***, rice, maize, Chinese cabbage, soybean, cucumber, and watermelon**. For the details, see Figure 4.

The 248 RRs members from above seven species were classified into four subgroups, namely, type-A, type-B, type-C, and pseudo RRs (Figure 6). All type-A RRs show quite close relationship. They probably function as negative regulators of cytokinin signaling in *Arabidopsis* (Tran et al., 2010). These genes show an alternating distribution of monocots and eudicots, indicating that type-A RR genes might already have expanded before the monocot-eudicot divergence. Type-B RRs from these seven species could be divided into five subgroups, namely type-B I, II, III, IV, and V as previous studies (Hwang et al., 2002; Liu et al., 2014). types-B I subfamily contains RRs from these seven species, but types-B II, III, IV, and V RRs form exclusive clusters of monocots or eudicots respectively in the phylogenetic tree. In detail, types-B II and III subgroups only contain *Arabidopsis* and Chinese cabbage RRs, whereas types-B IV and V are exclusively occupied by RRs from monocotyledons. Interestingly, all cucumber type-B RRs belong to the type-B I RRs, and the other types-B subgroup RRs are probably lost during evolution. Similar phenomenon was also found in soybean (Mochida et al., 2010). The type-C RRs from the seven species have close relationship in phylogenetic tree. They were suggested as the oldest RRs and type-A RRs are likely to be evolved from type-C RRs by mutations in their promoters (Pils and Heyl, 2009). All the pseudo RRs from seven plant species could be divided into type-B PRRs and clock PRRs. Type B PRRs are closer to the type-B authentic RRs rather than to the clock PRRs, just like in previous phylogenetic analysis (Mochida et al., 2010; Liu et al., 2014). These type B PRRs are likely to be evolved from the type-B RRs.

### Genomic distribution and gene duplication of TCS members

**Figure 7:**
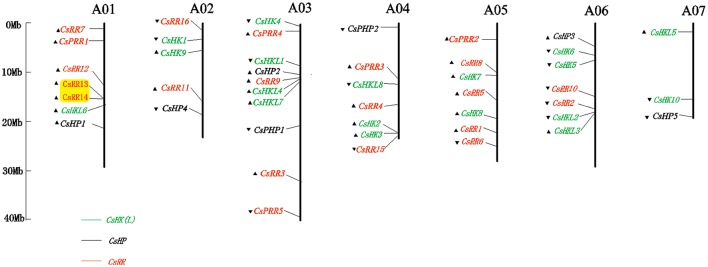
**Graphical representation of locations for putative TCS genes on cucumber chromosomes**. The chromosome number is indicated at the top of each chromosome. The arrows represent indicate the sense and antisense strands. The pairs of genes with tandem duplication have been highlighted.

The local distribution of TCS elements was also explored by blast search on the Cucurbit Genomics Database (Figure 7). The 46 cucumber TCS genes were apparently unevenly distributed on the seven chromosomes. In detail, the 18 putative CsHKs were scattered throughout the seven chromosomes. The CsRRs and CsHPs were mapped on all the cucumber chromosomes except for Chr7 and Chr5, respectively. Similarly, 49 TCS genes in watermelon were non-randomly located on the watermelon genome (Figure S12). The ClHK genes were mapped on the 10 watermelon chromosomes except for Chr7. The ClHP genes were scattered on the Chr5, Chr6, Chr7, and Chr8, whereas the ClRRs were distributed on all the chromosomes, except for Chr2 and Chr3.

Gene duplication, including segmental and tandem duplication plays a critical role in the expansion of TCS genes in several plant species (Hwang et al., 2002; Mochida et al., 2010; Liu et al., 2014). Ten pairs of segmental duplicates were found in *Arabidopsis*, which accounted for 35.71% of all *Arabidopsis* TCS genes. In Chinese cabbage, 61 out of 85 TCS genes were identified to be involved in segmental duplication. However, tandem duplication was not found in TCS genes from *Arabidopsis* and soybean, and only a pair of duplicate genes was identified in Chinese cabbage. These results suggested that segmental duplication was the main mechanism contributing to the duplication of TCS genes in *Arabidopsis*, Chinese cabbage, and soybean (Hwang et al., 2002; Mochida et al., 2010; Liu et al., 2014). Only three pairs (*CsRR13* and *CsRR14, ClHKL2* and *ClHKL3*, and *ClRR16* and *ClRR17*) of tandem duplications and one couple segment duplicate (*ClHP1* and *ClHP2*) were found in cucumber and watermelon, respectively (Figure 7, Figure S12). Previous studies have proved that cucumber and watermelon genomes probably do not undergo recent whole-genome duplication event (WGD). The absence of WGD might well explain why TCS genes in the two Cucurbitaceae crops lack gene duplication event, as well as contribute to the quite small TCS family size compared with other plant species such as *Arabidopsis*, rice, Chinese cabbage, and soybean (Hwang et al., 2002; Pareek et al., 2006; Mochida et al., 2010; Liu et al., 2014).

**Figure 8:**
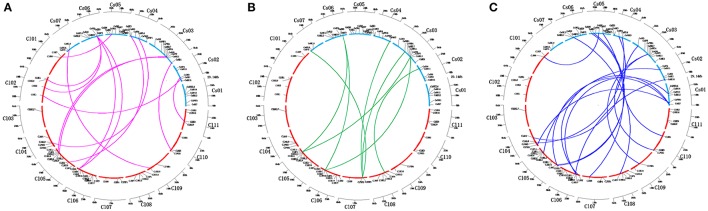
**Relationship diagrams of syntenic TCS genes in chromosomal synteny regions distribution among cucumber and watermelon**. Chromosomes of cucumber and watermelon are represented by blue and red arcs according to their own sizes. **(A–C)** Represent syntenic genes of HKs, HPs, and RRs in cucumber and watermelon are linked by red, green and blue lines respectively.

Cucumber and watermelon, which belong to the Cucurbitaceae family, have close phylogenetic relationship with each other. Almost all TCS proteins in cucumber share higher similarities with their counterparts in watermelon than other family members in cucumber. This result indicated that TCS elements remained highly conserved after the divergence of cucumber and watermelon. We further assessed the synteny relationships of TCS genes from cucumber and watermelon genomes using PGDD website to investigate cucurbit chromosomal evolution (Figure 8). A total of 42 syntenic gene pairs were found in HK(L)s, HPs, and RRs. The majority of cucumber TCS genes could be found in at least one syntenic gene in watermelon genome, except for the members from CsHKLs and type-B CsRRs. For the eight CsHKLs, only *CsHKL1, CsHKL7*, and *CsHKL8* in cucumber correspond to *ClHKL1, ClHKL9*, and *ClHKL8* in watermelon, respectively (Figure 8A). Meanwhile, only three type-B CsRRs, namely, *CsRR11, CsRR13*, and *CsRR16* have syntenic genes from watermelon (Figure 8C). These results indicated that these two subfamily members of cucumber might lose the synteny relationships with that in watermelon. Additionally, we also found that one synteny region of cucumber chromosome correspond to two or more regions from different chromosomes of watermelon, indicating the cross corresponding relationship of synteny regions between cucumber and watermelon (Figure 8). This result was consistent with comparative genomic analyses of cucumber, melon and watermelon genome that fusion and rearrangement events occurred in all cucumber chromosomes except Chr7, during chromosomal evolution (Huang et al., 2009).

The synonymous substitution rates (*Ks*) and non-synonymous substitution rates (*Ka*) were measured to explore the gene divergence mechanism between the two genomes. The *Ks* of 19 orthologous gene pairs between cucumber and watermelon were calculated to speculate the main divergence time of these TCS genes pairs between cucumber and watermelon (Figure S13). The *Ks* distribution peak from 0.15 to 0.25, which is consistent with that of the mean *Ks* distribution of 13,935 syntenic gene pairs from the genomic block of cucumber and watermelon in PGDD website. The *Ks* distribution suggested that the two genomes were diverged from 19.2 to 11.5 Mya, which is consistent with the previous hypothesis that watermelon diverged from cucumber at ~16 Mya ago (Lynch and Conery, 2000; Garcia-Mas et al., 2012).

The evolutionary patterns and gene duplication mechanism in cucumber and watermelon were further explored. The *Ks* of four gene pairs involved in tandem duplication (*CsRR13*/*CsRR14, ClHKL2*/*ClHKL3*, and *ClRR16*/*ClRR17*) and segment duplication (*ClHP1* and *ClHP2*) were analyzed. Their *Ks* were 0.4564, 1.4117, 0.3716, and 1.5518 corresponding to divergence times of 35.11, 108.59, 28.58, and 119.37 Mya, respectively. These duplication events occurred before the divergence of cucumber and watermelon, which confirmed recent WGD was absent and only a few segmental duplication events happened in the cucumber and watermelon genomes (Huang et al., 2009; Li et al., 2011; Guo et al., 2013).

### *Cis*-elements in putative promoter region of TCS genes in cucumber and watermelon

For predicting the potential functions of TCS genes in responses to stresses and hormones, 1.5 kb DNA sequence upstreams of the transcriptional start site of cucumber and watermelon TCS genes were downloaded from Phytozome and then used as queries to identify *cis*-elements on CARE website (Tables S4, S5). A considerable number of stress- and hormone-responsive *cis*-elements were identified. Notably, some drought- and ABA-responsive *cis*-elements such as MBS and ABRE, were identified in promoter region of 25 out of 46 TCS genes, whereas MeJA (CGTCA/TGACG-motif)-, salicylic acid (SA; TCA-element)-, and heat stress (HSE)-responsive elements were identified in 22, 20, and 22 promoters of cucumber TCS genes, respectively (Table S4-4). However, few studies have been reported on the roles of TCS genes involved in MeJA, SA, and heat stress responses (Firon et al., 2012). The *cis*-element analysis of TCS will help predict gene putative functions in various stress responses.

### Expression profiles of cucumber TCS genes in various tissues and during early fruit development

**Figure 9:**
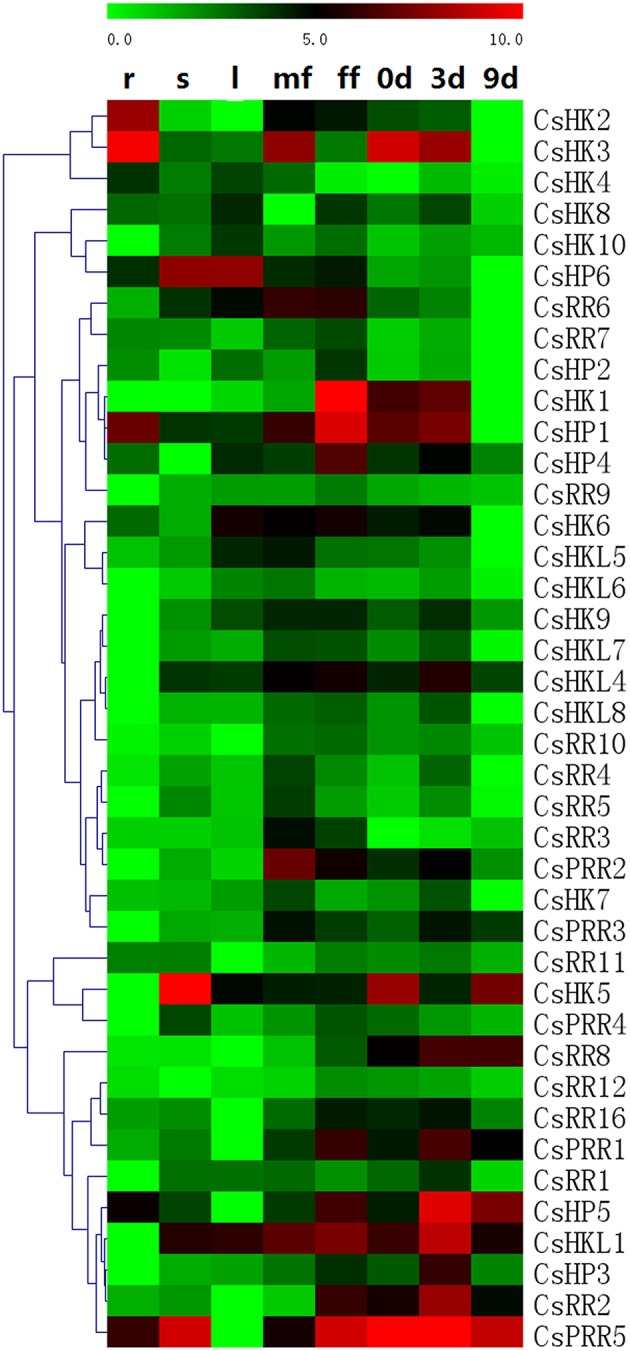
**Heat map representation for tissue- or organ-specific expression and fruit development–related expression profiles of TCS genes in cucumber**. The heatmap was manufactured by MeV4.8. The expression levels of genes are presented using fold-change values transformed to Log_2_ format compared to control. The Log_2_ (fold-change values) and the color scale are shown at the top of heat map. Green represents low expression, black represents medium expression, and red represents strong expression. R, roots; S, stems; L, leaves; mf, male flowers; ff, female flowers; 0 day: the fruits collected from the flower of anthesis, 3 day: Fruits from 3 days after pollination (DAP), 9 day: Fruits from 9 DAP.

We performed qRT-PCR to detect the expression patterns of 40 cucumber TCS genes in different cucumber organs/tissues (Figure 9, Table S6). Some detected genes such as *CsHK2*-*CsHK4, CsHP1*, and *CsPHP2* were relatively highly expressed in cucumber root. Especially, the transcript of *CsPHP2* was only detected in the root. Similar expression characteristics were reported in *Arabidopsis* and Chinese cabbage (Liu et al., 2014). But almost all of cucumber TCS genes expressed highest in fruit, male or female flowers. In detail, most of genes namely, *CsHK1/7/9, CsHKL5-8, CsHP1/2/4, CsRR3-7, CsRR9/10/12*, and *CsPRR2/3* were detected to express highest in male or female flowers, and they generally also have relatively high transcript levels in fruit at 3 DAP. But were hardly detected in root or 9 DAP fruit. Some genes such *CsHKL1/4/8, CsHP3/5, CsRR1/2/7/11/16*, and *CsPRR1/5* expressed highest in fruit and they have similar expression patterns in fruit, peaked at 3 DAP fruit and then decreased at 9 DAP fruit during early fruit development. And these genes usually have higher mRNA levels in female flower than male flower, and are expressed at low level in root or leaves. CsHK1, which was homologous with *Arabidopsis* CKI1, was expressed almost exclusively in the female flower. Similarly, *CKI1* is specifically expressed in *Arabidopsis* developing ovules and regulated female gametophyte development (Deng et al., 2010). Meanwhile, the mRNA levels of three ethylene receptors are relatively high in cucumber flower and fruit, and they have been verified to participate in the formation and development of female flower (Yamasaki et al., 2000, 2003; Wang et al., 2010). TCS members in *Arabidopsis* play important roles in regulating female gametophyte and fruit development (Marsch-Martínez et al., 2012). Similarly, some rice TCS genes are expressed predominantly in seeds compared with vegetative organs (Jain et al., 2008). Soybean *GmHK01, GmHP02, GmRR17*, and *GmRR34* are especially expressed in inflorescence or seed tissues (Mochida et al., 2010). Some tomato TCS elements were verified to play important roles in regulation of tomato fruit ripening (Tieman et al., 2000; Pan et al., 2013; Gupta et al., 2014). These data suggested that TCS genes probably play significant roles in cucumber reproductive development.

### Expression profiles of TCS genes in response to ZT and ABA

**Figure 10:**
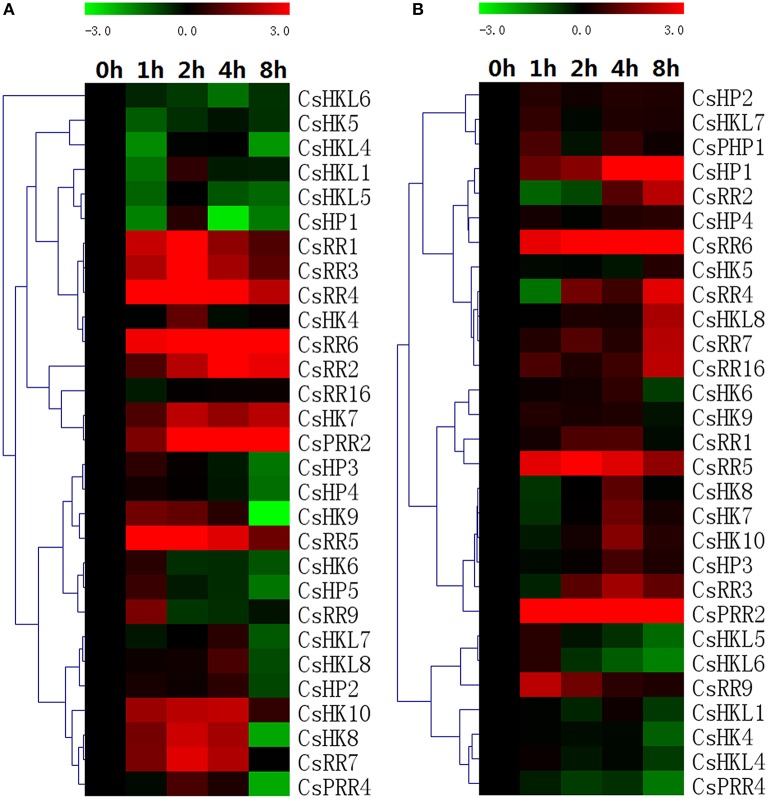
**Heat map representation for the expression patterns in response to exogenous ZT (A) and ABA (B) of TCS genes in cucumber**. Cucumber leaves were collected at 0, 1, 2, 4, and 8 h after 100 μM trans-zeatin or 100 μM ABA treatment. The heatmap was manufactured by MeV4.8. The color scale representing the relative expression values is shown in the upper left of the heatmap expressed in log2 scale.

The expression changes of 35 TCS members in cucumber were detected by real-time PCR analysis under the exposure of exogenous hormones. Almost all of the detected TCS genes markedly responded to exogenous ZT treatment (Figure 10A). Type-A RRs, *CsRR1*-*CsRR7*, were markedly upregulated by cytokinin especially from 2 to 4 h, whereas the expression of most type-B CsRR genes was modestly induced by ZT treatment. Similar response patterns were also found in *Arabidopsis* and Chinese cabbage (Rashotte et al., 2003; Tran et al., 2010; Liu et al., 2014). By contrast, all of CsHPs were notably downregulated at 8 h after treatment.

ABA is an extremely important hormone involved in regulating various stress- and development-related processes. Many plant TCS genes participate in regulating drought and salt via interacting with ABA (Tran et al., 2007, 2010; Wohlbach et al., 2008; Jeon et al., 2010; Pham et al., 2012). Here, most of detected cucumber TCS genes were generally upregulated by ABA except that *CsHK2, CsPHP1*, and *CsRR2/3* were downregulated, whereas some genes such as *CsRR8* and *CsRR23* didn't respond to exogenous ABA (Figure 10B). Actually, our qRT-PCR results of most detected TCS genes were inconsistent with promoter analyses in ABA response and this inconsistency was found in some stresses (drought and cold) and other species (Mochida et al., 2010; Liu et al., 2014). Even though most of cucumber detected TCS genes could respond to ABA, the promoter analysis indicated that the ABA-responsive elements (ABRE) were only found in the promoter regions of 14 cucumber TCS genes. Meanwhile, the ABRE elements were identified in the promoters of some genes such as *CsHK8* and *CsHP3* but they only responded slightly to ABA treatment (Figure 10B). The inconsistent data between expression profiles and promoter analyses were also found in soybean and Chinese cabbage TCS genes (Mochida et al., 2010; Liu et al., 2014).

### Expression patterns of TCS genes in response to abiotic stresses in cucumber

**Figure 11:**
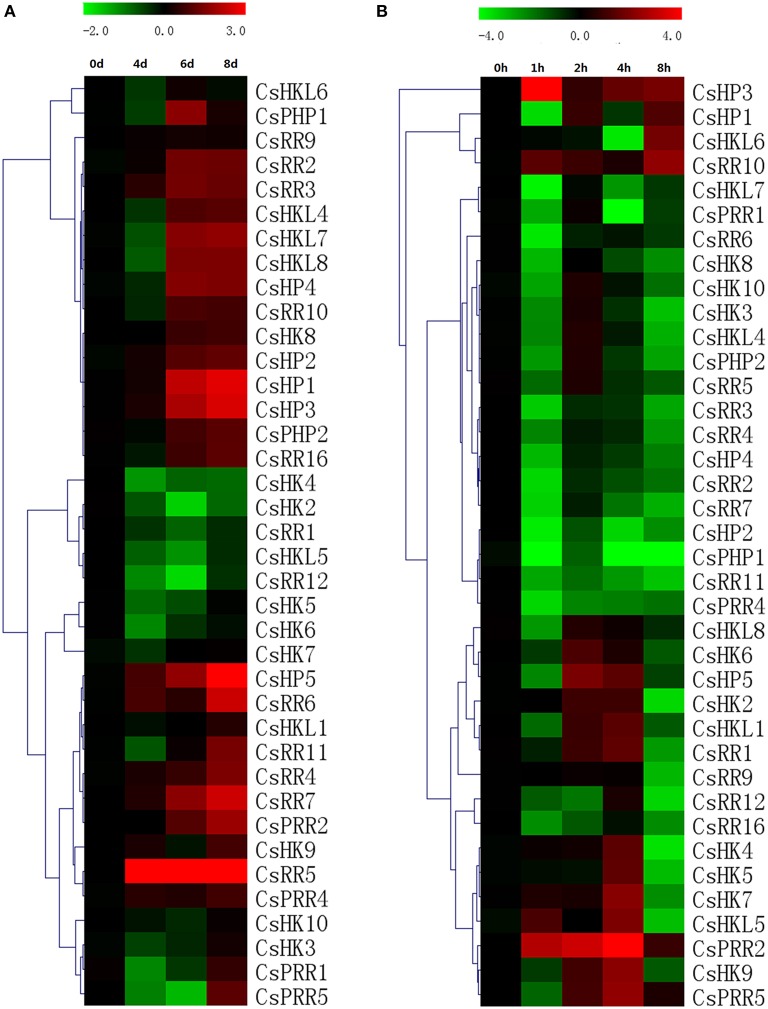
**Heat map representation for the response patterns to drought (A) and salt (B) stress of TCS genes in cucumber**. For drought treatment, the roots were collected from 4, 6, and 8 days after the seedlings withholding water. Cucumber roots were collected at 0, 1, 2, 4, and 8 h after salt treatment. For other details, see Figure 10.

**Figure 12:**
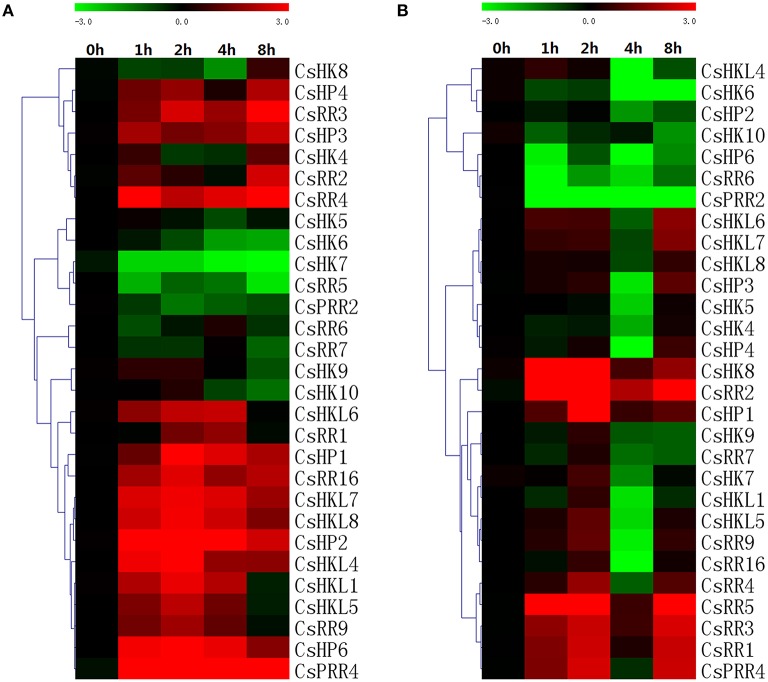
**Heat map representation for the response patterns to high (A) and low (B) temperature stress of TCS genes in cucumber**. Cucumber leaves were collected at 0, 1, 2, 4, and 8 h after the seedlings were moved to 35 ± 1°C for high-temperature treatment or 4 ± 1°C for low temperature treatment. For other details, see Figure 10.

TCS pathway is one of the most important mechanisms for stress signal transduction. In the present study, we found that all TCS genes detected in cucumber were induced by at least one abiotic stress, such as drought, salinity, heat, or low temperature (Figures 11, 12). However, they exhibited to be differentially regulated by these diverse abiotic stresses.

Roots are generally regarded as the main organs involved in drought and high salinity stresses and actually most of cucumber TCS genes expressed highly in root. For drought treatment, 38 out of 46 cucumber TCS genes were detected in root and most these detected genes such as *CsHP1, CsHP3, CsHP5, CsRR5, CsRR6*, and *CsRR7* were significantly upregulated. *CsHP5* mRNA level even increased up to 40-folds at 8 day after drought treatment as compared with controls (Figure 11A). However, some TCS members, such as *CsHK2-CsHK7, CsRR1, CsRR12*, and *CsHKL5* were downregulated by drought and their transcript levels declined to less than 30%. TCS genes in different species had diverse expression patterns in response to drought stress. A majority of *Arabidopsis* type-A ARRs were significantly upregulated by drought, whereas most of rice type-A RRs were down regulated (Kang et al., 2012). Similarity, most of TCS genes in Chinese cabbage were suppressed by drought stress (Liu et al., 2014). Most of the detected TCS transcripts were downregulated in response to salt stress in varying degrees (Figure 11B). The detected genes, except *CsHP1, CsHP3, CsHKL6, CsPRR2*, and *CsPRR5*, were evidently downregulated at 8 h after treatment. The expression levels of *CsHK2, CsHK4, CsPHP1*, and *CsRR12* even decreased over 10-fold. *CsPRR2* was exceptionally upregulated to nearly 60-fold at 4 h after high salt treatment.

In cucumber, almost all detected TCS genes could be upregulated or downregulated by cold stress (Figure 12A). In *Arabidopsis*, cold stress strongly induced the expression of type-A ARRs (Jeon et al., 2010). Similarly, type-A RRs-like genes in cucumber, *CsRR1-CsRR4*, were obviously induced by cold from 3- to 34-fold. Cucumber *CsHKL4* could be obviously induced by cold stress and play important roles in the development of cold tolerance (Sysoeva et al., 2013). Compared with the responses to cold treatment, most TCS genes were downregulated and reached the lowest expression levels at 4 h after heat treatment (Figure 12B), even some genes such as *CsHK6, CsHKL4, CsHP2*-*CsHP5*, and *CsPRR2* were downregulated to over 10-fold. But type-A RRs (*CsRR1-5*) were obviously induced except *CsRR4* was repressed at 4 h. In cucumber, *CsHP1, CsHP2*, and *CsHP3* were all homologous with *AHP1* but showed different expression profiles. *CsHP1* was obviously upregulated by 11-folds at 2 h after high-temperature treatment. However, the expression of other HPs (*CsHP2*-*CsHP5*) was markedly downregulated in response to heat stress. Consistently, *Arabidopsis AHP1, AHP2*, and *AHP3* genes are markedly downregulated by heat stress (Miyata et al., 1998). Meanwhile, promoter analyses showed that numerous heat stress-responsive elements (HSE elements) were identified in the putative promoter regions of TCS genes. Cucumber TCS genes are probably to be very important in response to heat stress, but studies on TCS members involved in heat stress are very limited (Firon et al., 2012).

## Conclusions

We identified 46 [18 HK(L)s, 7 HPs, and 21 RRs] and 49 [19 HK(L), 6 HPs, and 24 RRs] TCS genes in cucumber and watermelon, respectively. A comparative analysis of diverse plant species revealed that TCS members from the seven aforementioned species showed significant sequence and domain conservation. Gene duplication events rarely occurred in cucumber and watermelon TCS genes compared with those of *Arabidopsis*, Chinese cabbage, and soybean (Hwang et al., 2002; Mochida et al., 2010; Liu et al., 2014), which might be attributed to the absence of WGD in cucumber and watermelon genomes (Huang et al., 2009; Guo et al., 2013). Synteny analysis suggested that synteny regions from cucumber and watermelon chromosomes exhibited cross corresponding relationships. The majority of cucumber TCS genes were preferentially expressed in the early development of fruits and probably closely related to fruit development. Additionally, cucumber TCS genes could be upregulated or downregulated by various abiotic stresses and plant exogenous hormones. Our results have provided useful information by identifying candidate stress-responsive TCS genes for further functional analysis in cucumber.

## Author contributions

YH performed the experiments, analyzed the data, and drafted the manuscript. XL participated in qRT-PCR experiments and data analysis. TZ and CP collected the public dataset and assisted with data analysis, LQ and LC prepared the cucumber samples, GL conceived the study and its design and assisted with revisions to the manuscript. All authors read and consented to the final version of the manuscript.

### Conflict of interest statement

The authors declare that the research was conducted in the absence of any commercial or financial relationships that could be construed as a potential conflict of interest.
