# Supplementary material for: Genome-Wide Identification of Two-Component System Genes in Cucurbitaceae Crops and Expression Profiling Analyses in Cucumber
Source: Front Plant Sci. 2016 Jun 22;7:899. doi: 10.3389/fpls.2016.00899 (PMC4916222; doi:10.3389/fpls.2016.00899)
Supplement: Supplementary file 6 [file Table6.DOC]

**Table S6 Gene-specific primers in qRT-PCR analysis on TCS gene expression**

| **Primer name** | **Primer sequences** |
| --- | --- |
| EF1a-S | ACTGTGCTGTCCTCATTATTG |
| EF1a-A | AGGGTGAAAGCAAGAAGAGC |
| CsHK1-S | CACGCATTAGCAACGACAAG |
| CsHK1-A | CCTTGCTCTTACTTTGACATTG |
| CsHK2-S | GGCCAAGGAATCAGAGCTG |
| CsHK2-A | CTCAGCCATACTAACCACTCC |
| CsHK3-S | CAGTTATGGATGGGCTTGAAG |
| CsHK3-A | CCCAACTGCCTGTTTCCTC |
| CsHK4-S | GGGCTACTTACTTGCTACTTCG |
| CsHK4-A | CGTCGTCCTGGGAATCAAC |
| CsHK5-S | CTGATTTTACCGATACTGGGC |
| CsHK5-A | TTGATAGCTGTCCAAGGAGG |
| CsHK6-S | TTGGTGTCATTGATAAACGAGG |
| CsHK6-A | TGATGATTTGCCTAAACCTTCC |
| CsHK7-S | GGGAGTTGAGTTGGCAGTATTC |
| CsHK7-A | CAGAGTTACCCACGAGATTTG |
| CsHK8-S | CAGGAGAAGGCAAGTGGC |
| CsHK8-A | CTCAAAGGGTTTCGAGACATAG |
| CsHK9-S | CACTTGCATACTTCTCGATCC |
| CsHK9-A | AGAACAATGAAAGCACCAAAC |
| CsHK10-S | GATGTTGTCGCAGACCAGG |
| CsHK10-A | CCAAAAGCAGGGATGAAAGAG |
| CsHKL1-S | GAGGAACGCTTGAGTTTTACC |
| CsHKL1-A | GTTCAGATGATTCTCCAGGTTC |
| CsHKL2-S | GTTGACGGGTTACGATAGAGTG |
| CsHKL2-A | GAATGTCAGTGGCTGGATAATG |
| CsHKL3-S | GCATAGGGACAGATGTGAGGAC |
| CsHKL3-A | CCAAGATACGGCTCAAGACC |
| CsHKL4-S | TCAACAGGTCTTAGCCGATTTC |
| CsHKL4-A | CTAATCCTTCTTGAGTCACCCATC |
| CsHKL5-S | GTTGTATGATTGCTGTGGACG |
| CsHKL5-A | CTTCTTGCTGCTCGATGTTAG |
| CsHKL6-S | GCTCTATTTACGCCTTCCTCTG |
| CsHKL6-A | CATAAAACGGCTTCTGGGTAG |
| CsHKL7-S | CACTTACCTCTACACCACTGC |
| CsHKL7-A | CGAGCGTACAGACGACTTATTG |
| CsHKL8-S | CATAGTTGCTGATGGAATTGAGG |
| CsHKL8-A | GGCAGTGGTGTAGAGGTAAGTG |
| CsHP1-S | TTTAGTGAAGAACAAGTTGGAGAC |
| CsHP1-A | GTTACATATTGGATTTGAGGC |
| CsHP2-S | AGGCAGCAGTTCCAGCATAG |
| CsHP2-A | CACCAGCATTCAAAATCCG |
| CsHP3-S | GGAAGCAGTTCAAGCATAGG |
| CsHP3-A | CAATTTGTTGCTCCATCCTG |
| CsHP4-S | GAACTGCAGGATGATACTAACC |
| CsHP4-A | TGAGCTACTACCCTTGAACTGG |
| CsPHP1-S | GTAACTTTATGCTACCGTGATTC |
| CsPHP1-A | CCACTTCCAGCCTTAATGTATTC |
| CsHP5-S | AAGGCAGGAAGTGGAGAAGG |
| CsHP5-A | GTCTTGCGAGCTGAAAATAGG |
| CsPHP2-S | GCCAATTCTGAACAAAACAAC |
| CsPHP2-A | TAACTCCTGCTCCCAAAACTC |
| CsRR1-S | GCCTCCTTCGTCTTACTGC |
| CsRR1-A | CTTCAACCCATCAAACCCAAC |
| CsRR2-S | GATTTCTGTATGCCTGGGATG |
| CsRR2-A | CATGTTGATTCTCGATGGCTC |
| CsRR3-S | CATCGCTAAGAAACATACCAGTG |
| CsRR3-A | GTTTCAGTTTATTCACATCGCAC |
| CsRR4-S | AAACCTCCTCTTACCAAGTCAC |
| CsRR4-A | CTTCAACAAATCATAGCCAGTC |
| CsRR5-S | AGCAAACGGACTCCAAAATC |
| CsRR5-A | GCTCCTTCTTCCAAGCATCTG |
| CsRR6-S | AACTTCCTCCTTCAATGTTACTG |
| CsRR6-A | TGTCATCTCTGGCATACAATAATC |
| CsRR7-S | TATGGAAGGAGAAAATGGAGG |
| CsRR7-A | TGAATGTATGATCGAGGACGG |
| CsRR8-S | AATGAGTGGCAGCAGAGGTC |
| CsRR8-A | GATTGTTTCAAGGGTTTCAGC |
| CsRR9-S | AGATGCTTCGTATTTGCCG |
| CsRR9-A | CACCATGAGTCACTCCCTTC |
| CsRR10-S | TGGGAACTTCATACAGGATAGC |
| CsRR10-A | TCTGAGATTTGACCTCCACAGG |
| CsRR11-S | CAACACGGTGCTTGTGATTATC |
| CsRR11-A | CCCCTGAGGAACTGGCTATC |
| CsRR12-S | TCATTCTGTTGCTGTAGTCGG |
| CsRR12-A | TGATATTGGCATTTACGGAGG |
| CsRR13-S | CAAGAGCTGAGGAAGCAATAAG |
| CsRR13-A | CCGACAGGTTTTGGTAAATAGAG |
| CsRR14-S | GTGTGCCTTCGTTATCTTGCTC |
| CsRR14-A | GCCATCCATGTATTGTCTGAC |
| CsRR15-S | GCTTGTTGGGCTGGAGATG |
| CsRR15-A | ACGAGGCTTCTTCTGGGTTG |
| CsRR16-S | ACCGAGAACCGTTAGACAGC |
| CsRR16-A | CAGGAGGGTAAATTCCAGTG |
| CsPRR1-S | ATTTTCACCATTGCCCTCC |
| CsPRR1-A | GCTCCTTCCTTTTCTGCC |
| CsPRR2-S | GACAAGCGTCATCCAATGTG |
| CsPRR2-A | TTCCTCTGAACTCCAGCAAC |
| CsPRR3-S | CATCTTAGGTCTCTCAAGGTTC |
| CsPRR3-A | TTCTTCCTCGTTTTATGGTTC |
| CsPRR4-S | TAGCCCTCTCTCTTCTCATCCTC |
| CsPRR4-A | TGCCTCTTTCACCACTTTGTC |
| CsPRR5-S | CAGAGAGAAGAAAATACAAGGTGG |
| CsPRR5-A | GTTTGGATGATAAGAAGGGTAAGC |
