# Supplementary material for: Genome-Wide Identification of Two-Component System Genes in Cucurbitaceae Crops and Expression Profiling Analyses in Cucumber
Source: Front Plant Sci. 2016 Jun 22;7:899. doi: 10.3389/fpls.2016.00899 (PMC4916222; doi:10.3389/fpls.2016.00899)
Supplement: Supplementary file 7 [file DataSheet1.docx]

***Supplementary Material***

# Genome-wide identification and expression analysis of two-component system genes in *Cucumis sativus* and *Citrullus lanatus*

## Yanjun He^1^, Xue Liu^1^, Tao Zou^1^, Changtian Pan^1,2^, Li Qin^1^, Lifei Chen^1,2^, Gang Lu^1,2^*

^1^Key Laboratory of Horticultural Plant Growth, Development and Biotechnology, Agricultural Ministry of China, Department of Horticulture, Zhejiang University, China

^2^Zhejiang Provincial Key Laboratory of Horticultural Plant Integrative Biology, China

*** Correspondence:**

Prof. Gang Lu, Department of Horticulture, Zijingang Campus, Zhejiang University, Hangzhou, 310058 Zhejiang, China

glu@zju.edu.cn

**Supplementary Figures and Tables**

**Supplementary Figures**

**
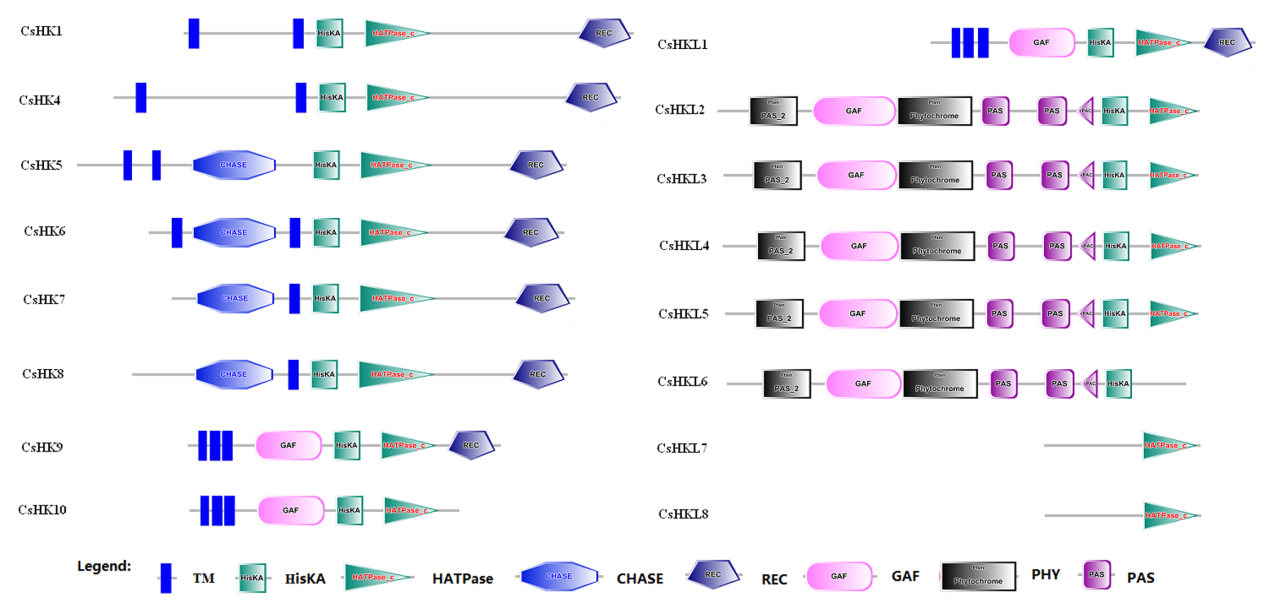
**

**Figure S1.** Domain structures of CsHK(L) proteins in cucumber. Domain structures were analyzed by SMART online tool and drawn according to their original location and size. TM, transmembrane region; HisKA, Histidine kinase domain; HATPase, Histidine kinase-like HATPases; Rec, receiver domain; CHASE, cyclase/histidine kinase-associated sensory extracellular domain; GAF, cyclic GMP adenylyl cyclase FhlA domain; PHY, chromophore-binding domain; PAS, Per/Arndt/Sim folds.

**

**

**Figure S2.** Amino acid sequence alignment of CsHK(L)s in cucumber. Histidine kinase (like) (A), receiver (B) and cyclase/histidine kinase-associated sensory extracellular (CHASE) (C) domains from CsHK(L) proteins in cucumber were aligned by the Clustal X program. The conserved motifs have been marked.

**
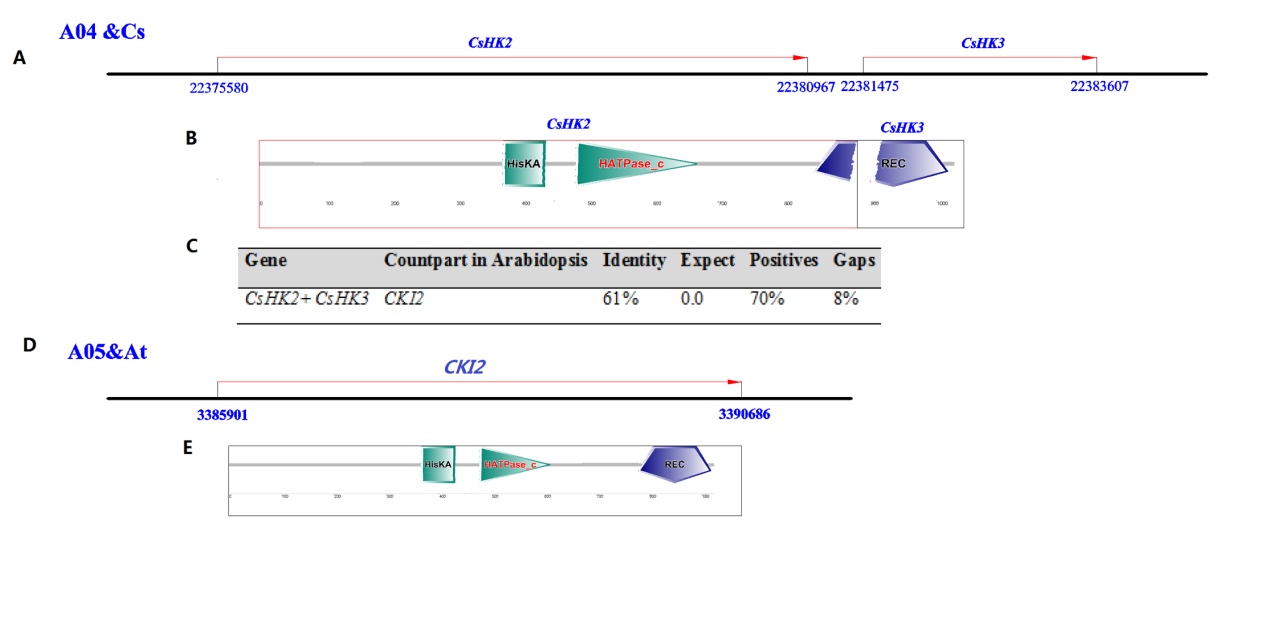
**

**Figure S3.** Conserved domain and similarity analysis of a pair of fractured genes (CsHK2 and CsHK3) and their homologous genes CKI2 in Arabidopsis. (A) Physical positions of the CsHK2 and CsHK3 on the chromosome of cucumber. (B) Conserved domains of CsHK2 and CsHK3. (C) Homologous analysis between CsHK2 and CsHK3 and CKI2. (D) Physical positions of the CKI2 on the chromosome of Arabidopsis. (E) Conserved domains of CKI2.


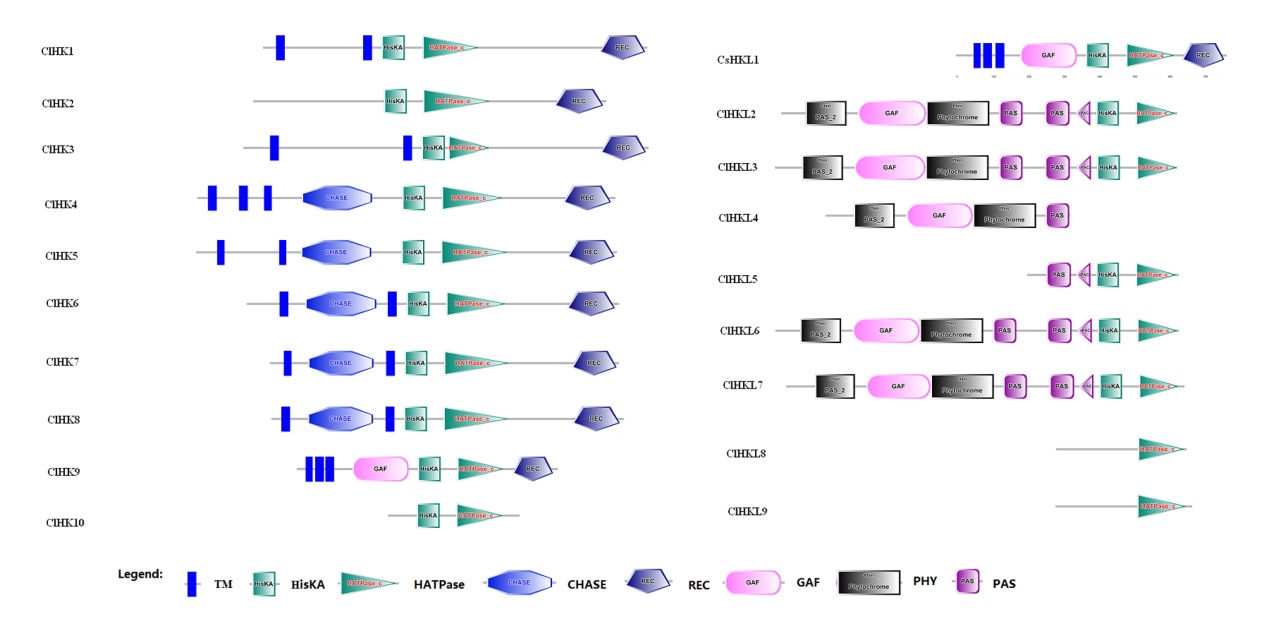


**Figure S4.** Domain structures of ClHK(L) proteins in watermelon. Domain structures were analyzed by SMART online tool and drawn according to their original location and size. For other detailes, see Supplementary Figure S1.


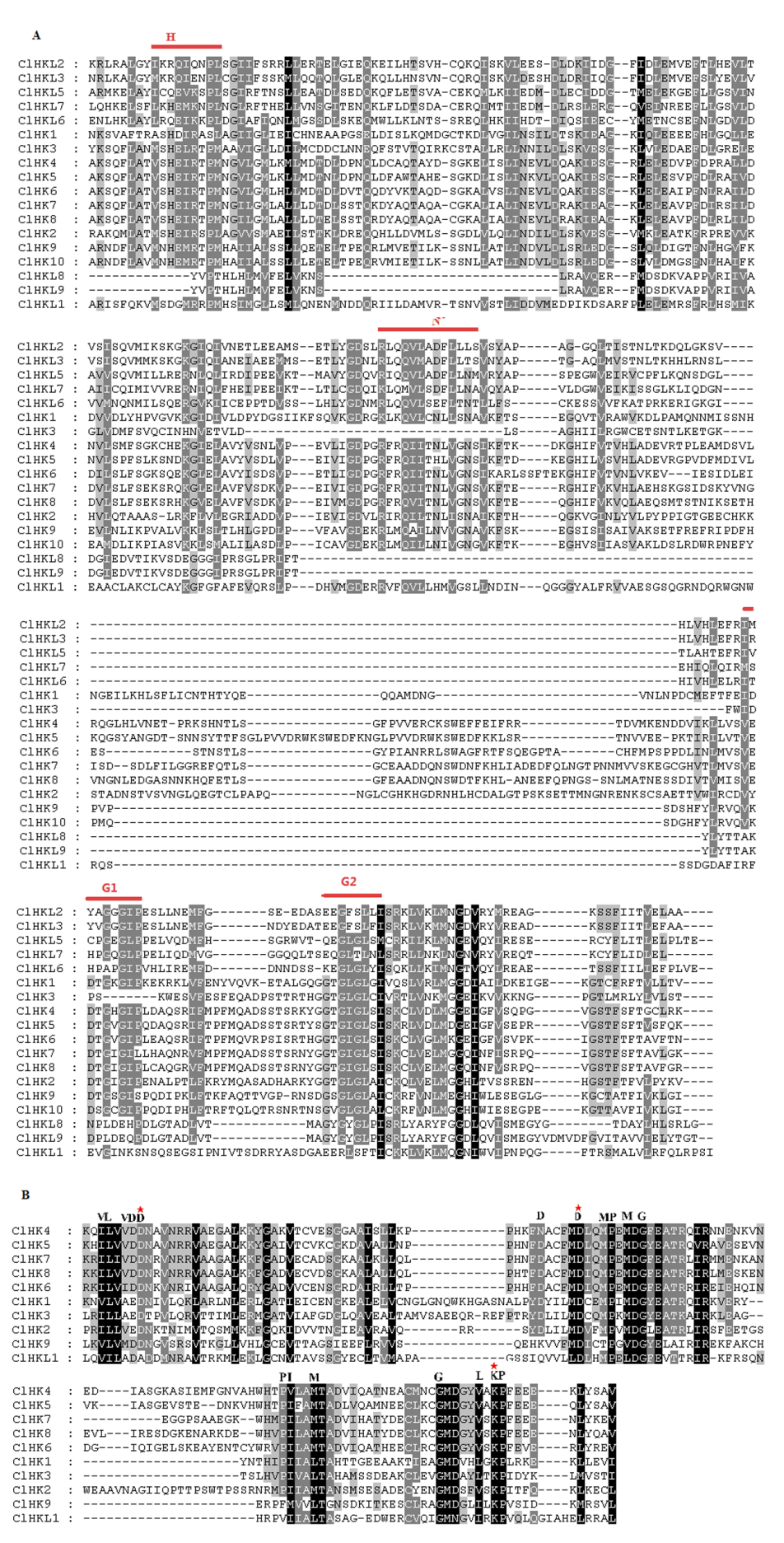


**Figure S5.** Amino acid sequence alignment of ClHK(L)s in watermelon. Transmitter (A) and receiver (B) domains of HK(L)s. Sequences were aligned by the Clustal X program. The conserved motifs have been marked.

**

**

**Figure S6.** Domain structures and alignment of deduced amino acid sequences of CsHP proteins in cucumber. (A) Domain structures were analyzed by SMART online tool and drawn according to their original location and size. HPt, His-containing phosphotransfer domain; PHPt, pseudo His-containing phosphotransfer domain. (B) Sequences were aligned by the Clustal X program. The Hpt domain has been highlighted by red line. The conserved XHQXKGSSXS motif was also marked above the alignment.


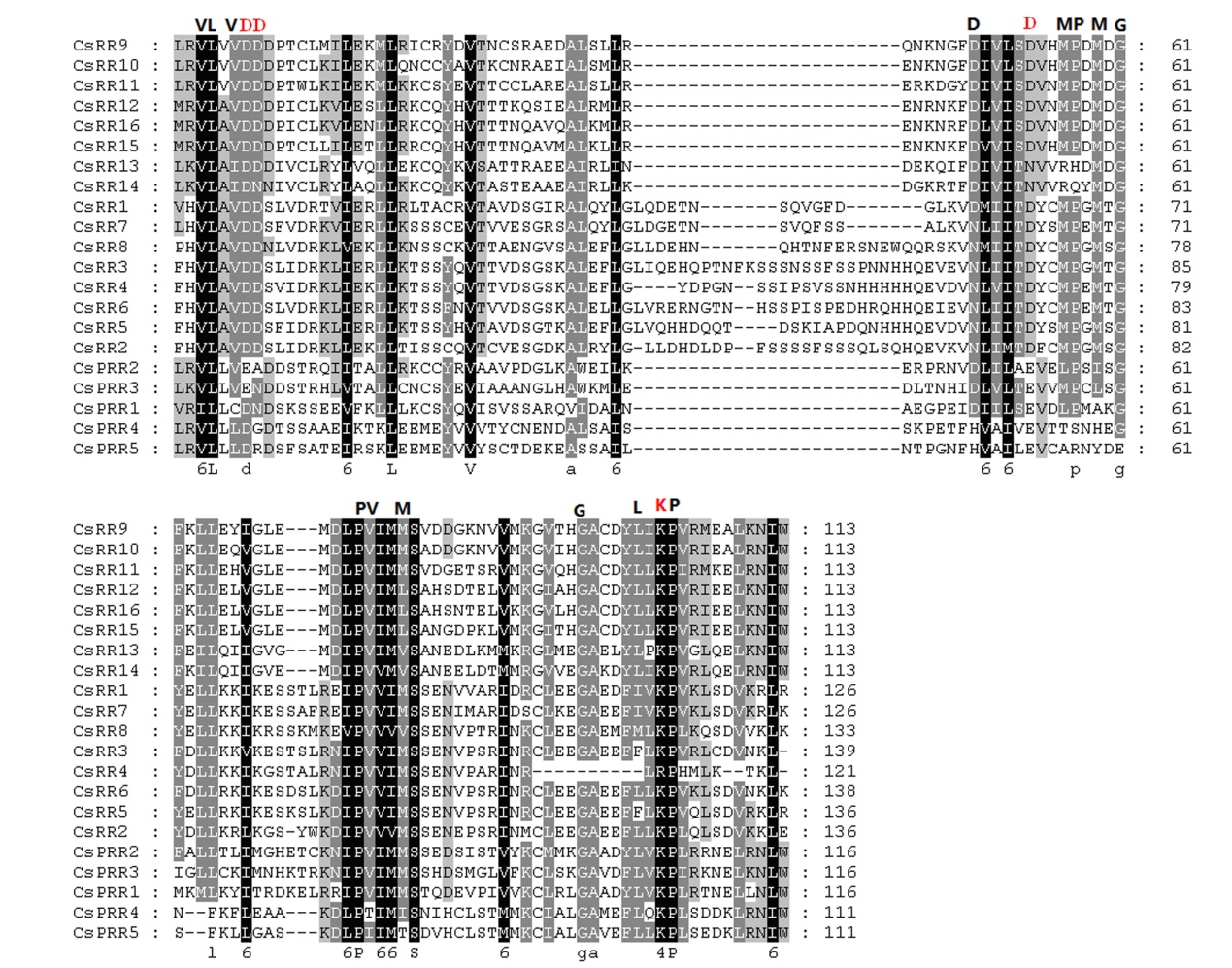


**Figure S7.** Alignment of deduced amino acids sequences of the Rec domain from RR proteins in cucumber. Sequences were aligned by the Clustal X program and the highly conserved amino acids are highlighted.


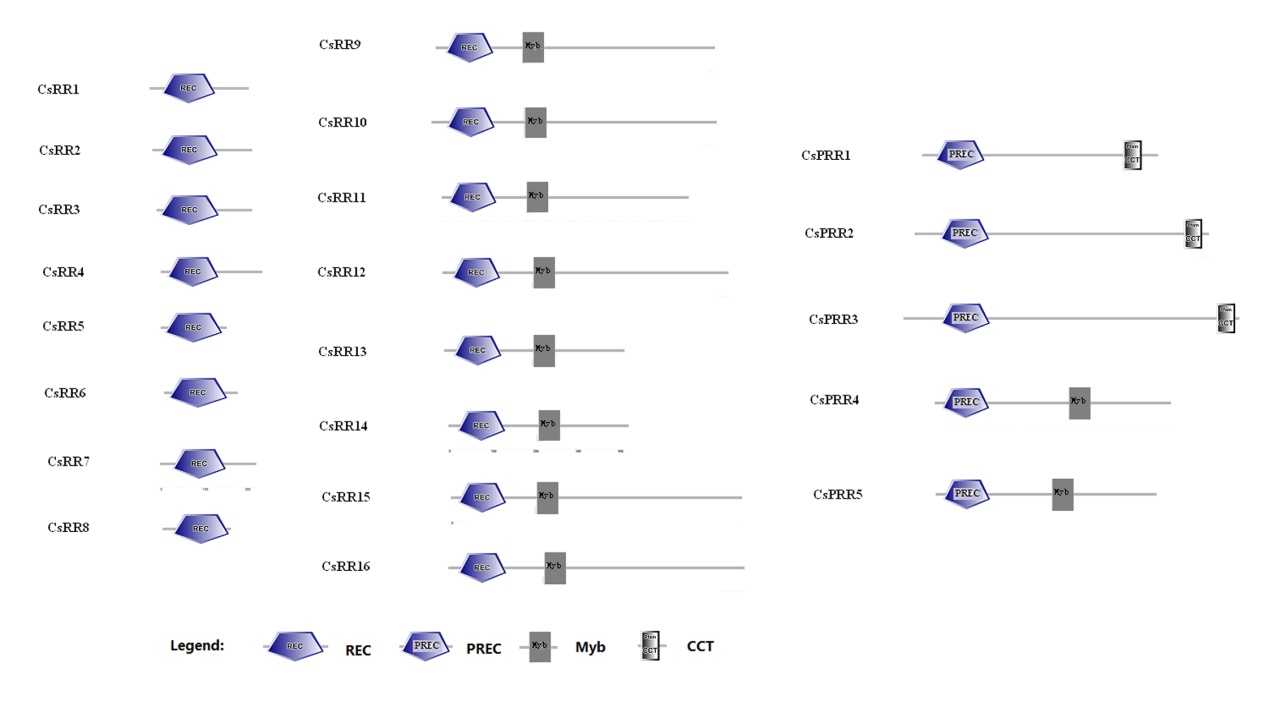


**Figure S8.** Domain structures of CsRR genes in cucumber. Domain structures were analyzed by SMART online tool and drawn according to their original location and size. Rec, receiver domain; PRec, pseudo receiver domain; Myb, Myb DNA-binding domain; CCT, plant-specific CCT motif.

**
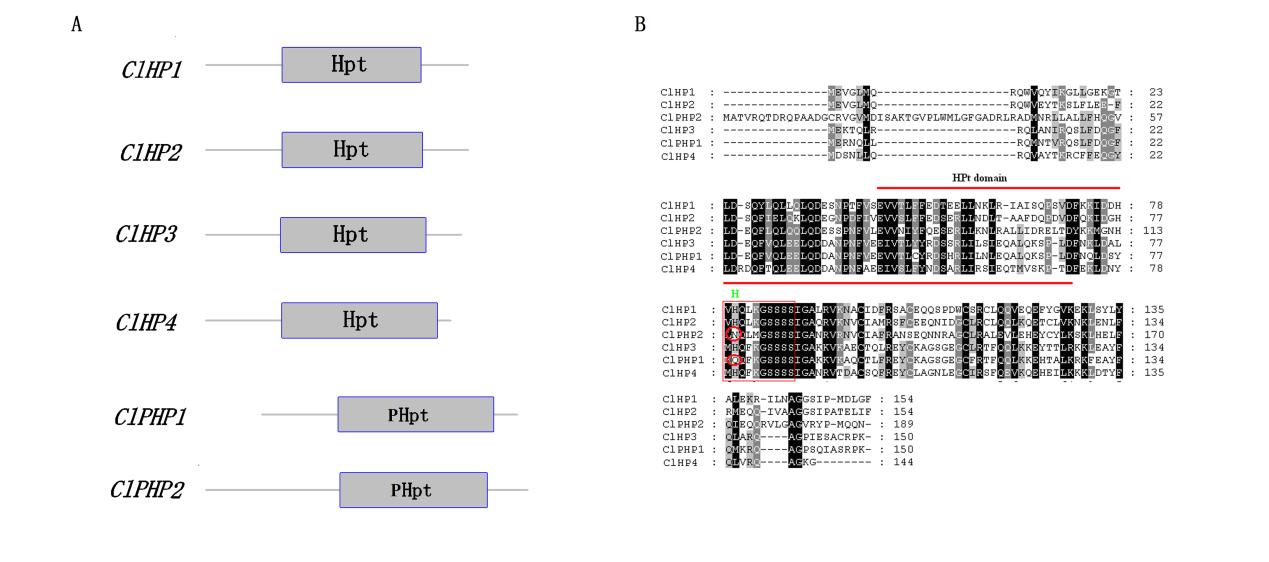
**

**Figure S9.** Domain structures and alignment of deduced amino acid sequences of ClHP proteins in watermelon. (A) Domain structures were analyzed by SMART online tool and drawn according to their original location and size. (B) Sequences were aligned by the Clustal X program. For other details, see Supplementary Figure S6.


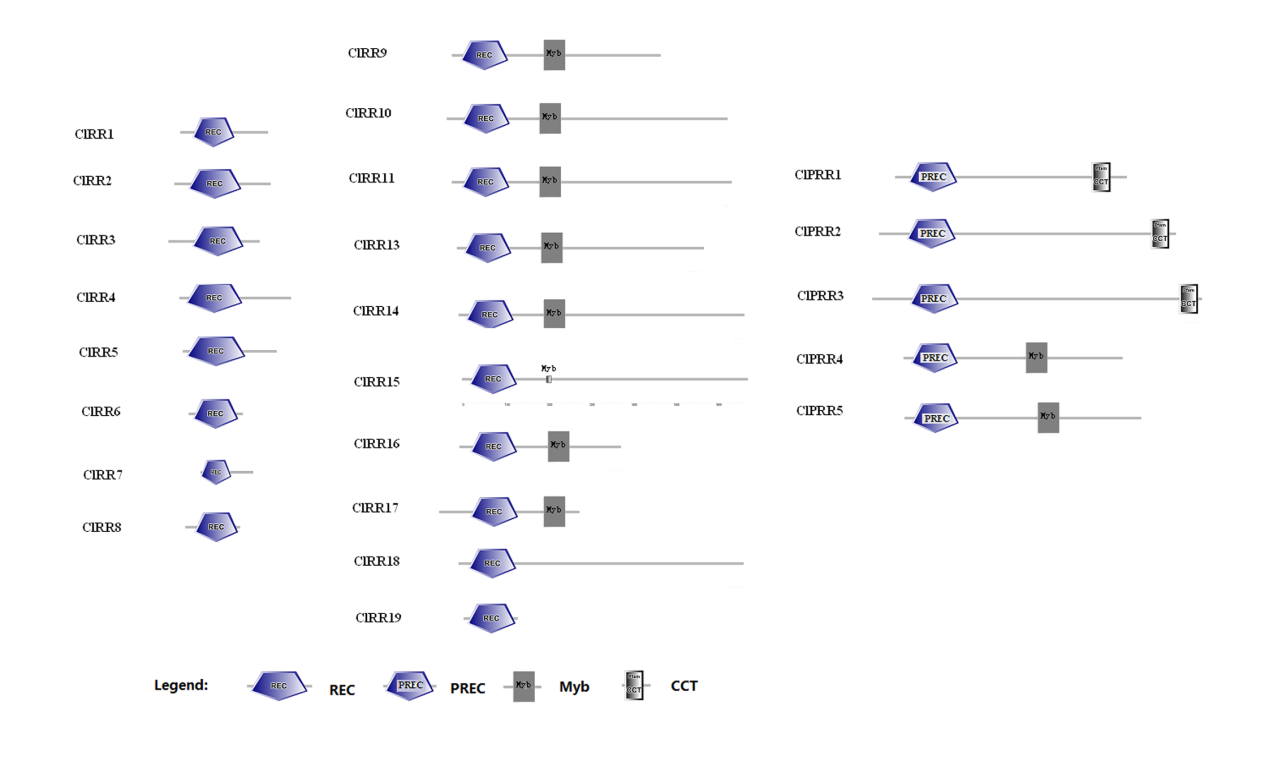


**Figure S10.** Domain structures of ClRR genes in watermelon. Domain structures were analyzed by SMART online tool and drawn according to their original location and size. For other details, see Supplementary Figure S8.


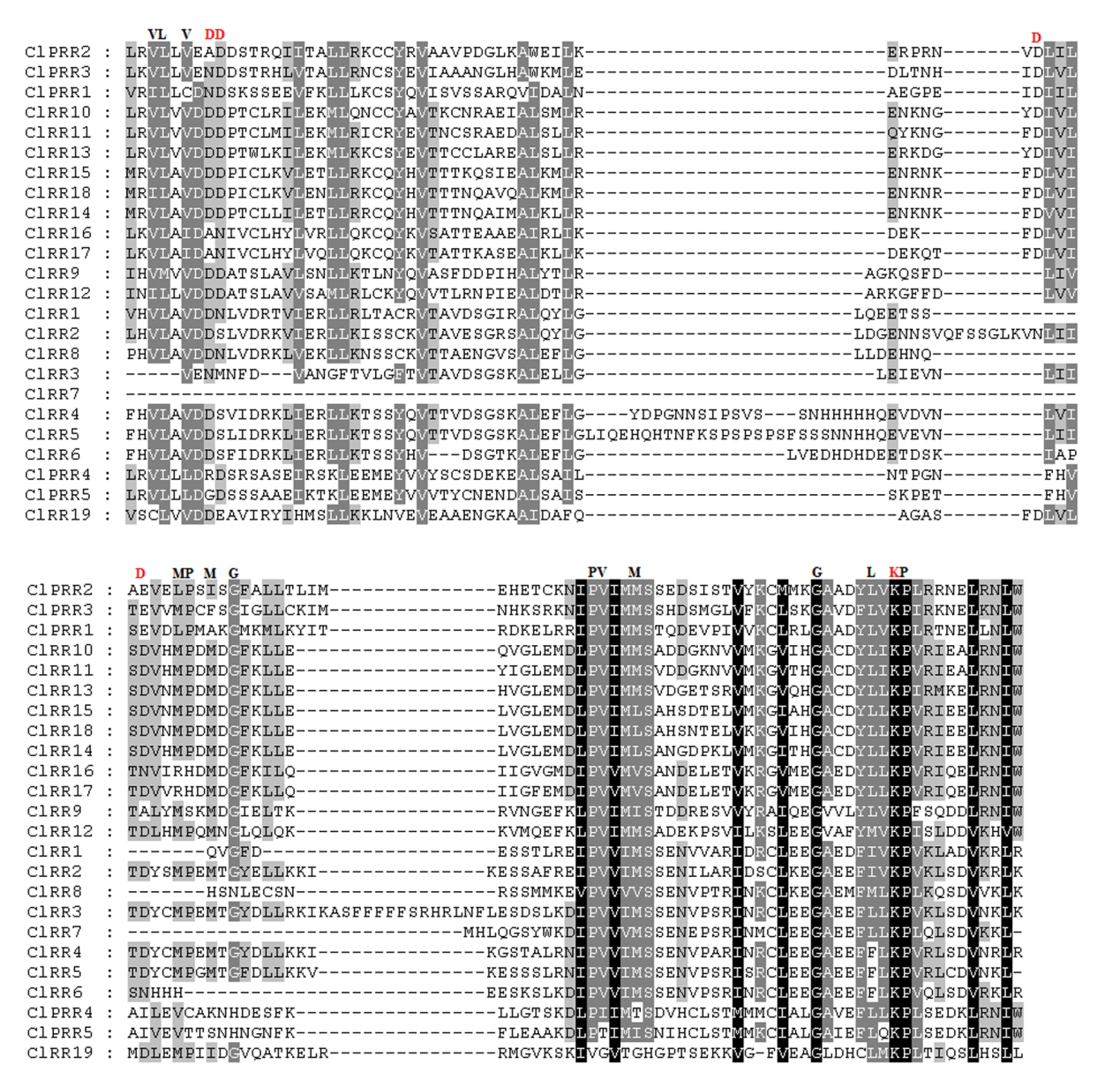


**Figure S11.** Alignment of deduced amino acids sequences of the Rec domain from RR proteins in watermelon. Sequences were aligned by the Clustal X program and the highly conserved amino acids are highlighted.


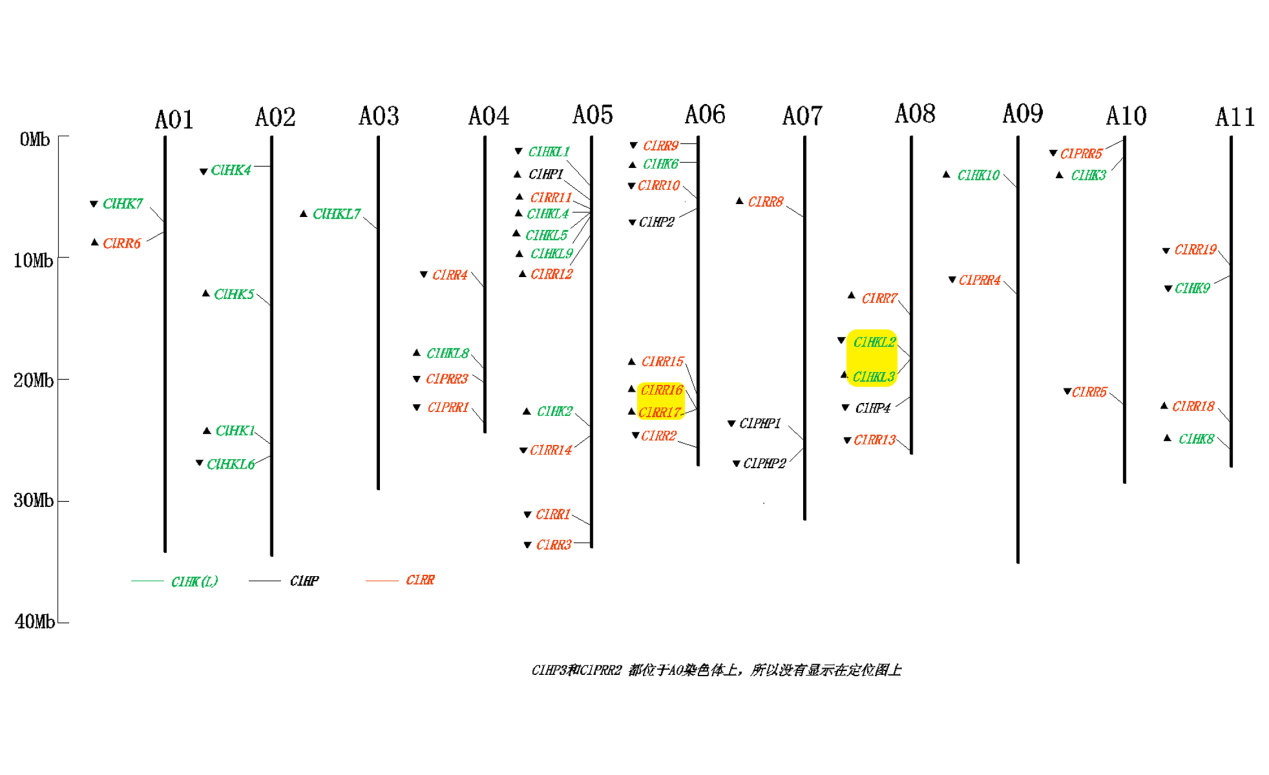


**Figure S12.** Graphical representation of locations for putative TCS genes on watermelon chromosoms. The chromosome number is indicated at the top of each chromosome. The arrows represent indicate the sense and antisense strands. The pairs of genes with tandem duplication have been highlighted.


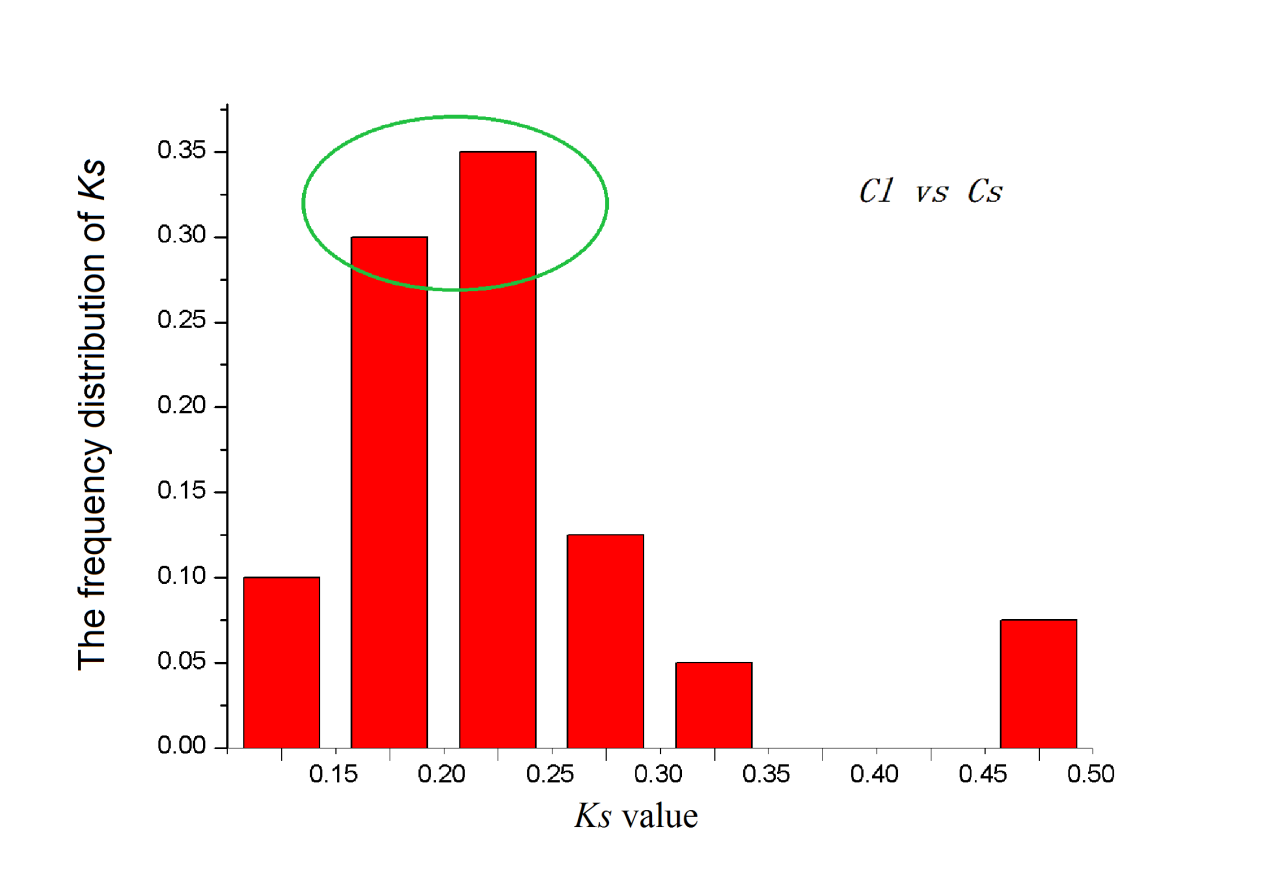


**Figure S13.** The *Ks* values distribution of the orthologous gene pairs within TCS genes between cucumber and watermelon. The *Ks* value distributions were obtained from duplicated paralogous genes pairs in cucumber and watermelon. The vertical axis indicates the frequency of paired sequences, while the horizontal axis represents the *Ks* values with a 0.05 interval.


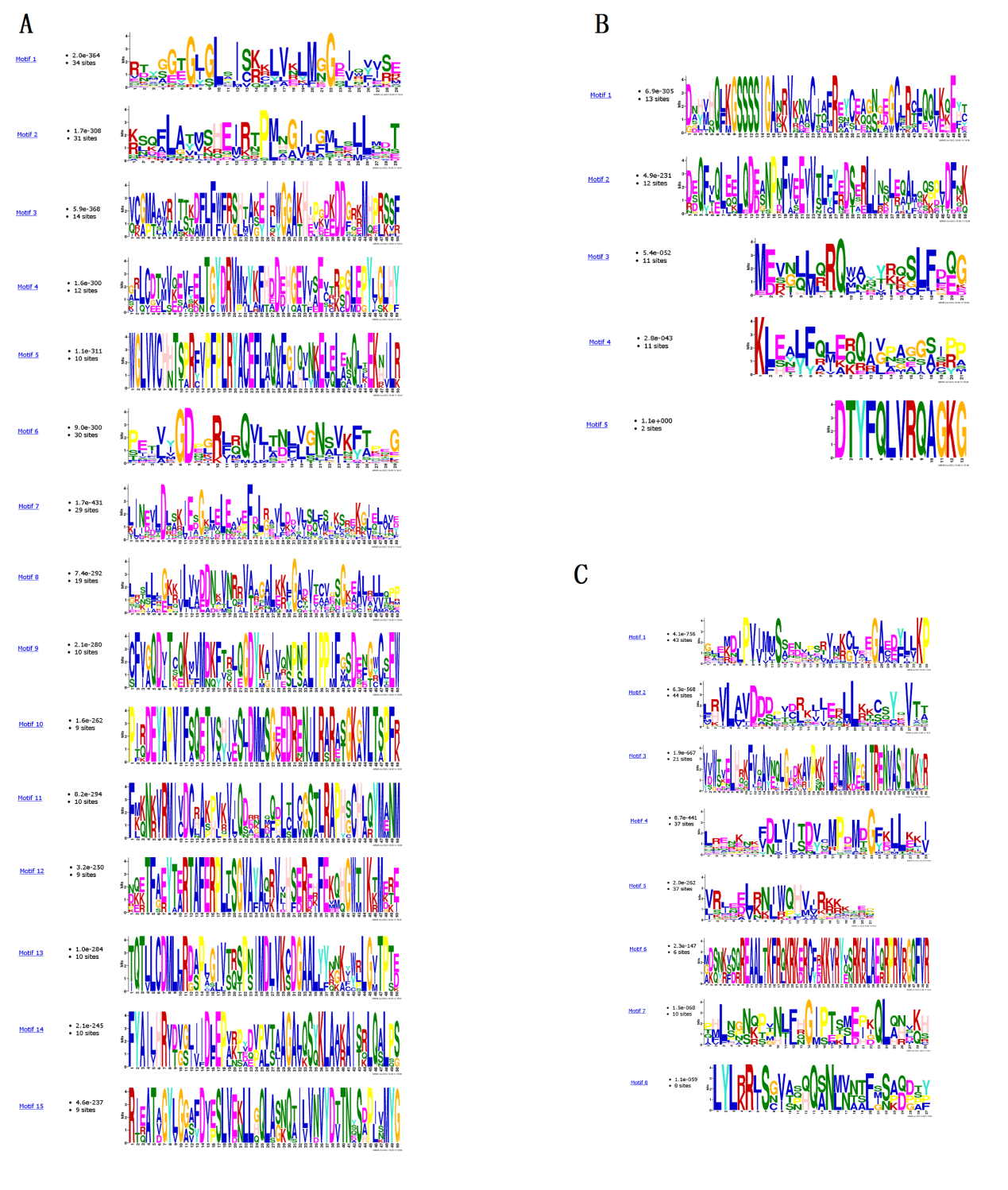


**Figure S14.** The conserved motif LOGO of HK(L) (A), HP (B), and RR (C) genes in cucumber and watermelon.
